# Chronic conditions and aortic disease risk: a prospective cohort study with predictive and etiological analyses

**DOI:** 10.1038/s41467-026-76551-y

**Published:** 2026-08-11

**Authors:** Linling Yu, Peijiang Lu, Meng Yang, Xiong Wang, Qi Liu, Pinpin Long, Ze-Min Fang, Zihao Zhang, Jinzhu Zhao, Qi Xiang, Canqing Yu, Huaidong Du, Ling Yang, Yiping Chen, Zhengming Chen, Jun Lv, Liming Li, Dianjianyi Sun, Wei Liu, Xin Yi, Ding-Sheng Jiang

**Affiliations:** 1 Department of Public Health, Tongji Hospital, Tongji Medical College, Huazhong University of Science and Technology, Wuhan, Hubei China; 2 Division of Cardiovascular Surgery, Tongji Hospital, Tongji Medical College, Huazhong University of Science and Technology, Wuhan, Hubei China; 3 Institute of Maternal and Child Health, Wuhan Children’s Hospital (Wuhan Maternal and Child Health Care Hospital), Tongji Medical College, Huazhong University of Science and Technology, Wuhan, Hubei China; 4 Department of Laboratory Medicine, Tongji Hospital, Tongji Medical College, Huazhong University of Science and Technology, Wuhan, Hubei China; 5 Department of Epidemiology & Biostatistics, School of Public Health, Peking University, Beijing, China; 6 Daxing Center for Disease Control and Prevention, Beijing, China; 7 Department of Occupational and Environmental Health, School of Public Health, Tongji Medical College, Huazhong University of Science and Technology, Wuhan, Hubei China; 8 Department of Cardiology, University Heart Center Zurich, University Hospital Zurich, Zurich, Switzerland; 9 Division of Child Healthcare, Department of Pediatrics, Tongji Hospital, Tongji Medical College, Huazhong University of Science and Technology, Wuhan, Hubei China; 10 Peking University Center for Public Health and Epidemic Preparedness & Response, Beijing, China; 11 Key Laboratory of Epidemiology of Major Diseases (Peking University), Ministry of Education, Beijing, China; 12 Clinical Trial Service Unit & Epidemiological Studies Unit (CTSU), Nuffield Department of Population Health, University of Oxford, Oxford, United Kingdom; 13 State Key Laboratory of Vascular Homeostasis and Remodeling, Peking University, Beijing, China; 14 Department of Cardiology, Renmin Hospital of Wuhan University, Wuhan, Hubei China; 15 Key Laboratory of Organ Transplantation, Ministry of Education, Chinese Academy of Medical Sciences, Wuhan, Hubei China; 16 NHC Key Laboratory of Organ Transplantation, Wuhan, Hubei China

**Keywords:** Risk factors, Vascular diseases, Epidemiology

## Abstract

Aortic diseases are often clinically silent until advanced stages, and risk determinants beyond traditional cardiovascular factors remain incompletely characterised. Here, we investigate associations of 42 chronic conditions and multimorbidity with incident aortic disease in UK Biobank (UKB), with external validation in China Kadoorie Biobank (CKB). In UKB, 21 chronic conditions are associated with overall aortic disease after false discovery rate correction, including coronary heart disease, hypertension, atrial fibrillation, peripheral vascular disease, heart failure, and COPD; similar patterns are observed in CKB. Aortic atherosclerosis shows the broadest comorbidity profile, whereas dissection and aneurysm subtypes show narrower profiles. Higher multimorbidity burden is associated with higher incidence in both cohorts. In UKB, hypertension shows the largest estimated population-attributable fraction, with additional contributions from COPD and chronic kidney disease. Mendelian randomization supports selected associations, and machine-learning models show internal discriminatory performance. These findings support consideration of multimorbidity in aortic disease risk assessment.

## Abstract (web summary)

This study shows that multimorbidity and several chronic conditions, including cardiovascular, respiratory and kidney diseases, are associated with higher risk of aortic disease in two large cohorts.

## Introduction

Aortic diseases, including aortic aneurysm, dissection and atherosclerotic disease of the aorta, are major causes of cardiovascular morbidity and premature death^1–3^. Although relatively uncommon in the general population, they are often clinically silent until presentation with rupture, malperfusion or acute dissection, at which point case fatality is high and opportunities for prevention are limited^4^. Contemporary clinical management, therefore, relies heavily on identifying individuals at increased risk before catastrophic events occur. Current guidelines emphasise imaging surveillance, family history and selected genetic syndromes, but risk stratification in the general population remains limited^5^.

Traditional risk factors for aortic disease, such as trauma, systemic arterial hypertension, and cocaine use, are well established^3^. However, populations worldwide are ageing, and the burden of chronic non-communicable diseases is rising; the World Health Organisation estimates that non-communicable diseases accounted for about three-quarters of non-pandemic-related deaths globally in 2021^6^. Multimorbidity, commonly defined as the coexistence of two or more chronic conditions, is now highly prevalent in adult and especially older populations, with substantial variation across settings but consistently increasing with age, affecting over one-third of adults globally^7^. In this context, multimorbidity may capture cumulative vascular, metabolic, inflammatory and haemodynamic stress that is not fully reflected by individual risk factors considered in isolation.

Yet the contribution of multimorbidity to incident aortic disease remains poorly resolved. Prior studies have largely focused on single disorders, specific aortic phenotypes, or selected high-risk clinical groups, leaving several important questions unanswered^8,9^. First, it is unclear whether chronic conditions show shared or distinct associations across clinically heterogeneous aortic disease subtypes, including thoracic aneurysm, abdominal aneurysm, dissection and aortic atherosclerosis. Second, the population-level burden attributable to multimorbidity has not been well quantified. Third, whether multimorbidity-related information improves risk prediction beyond routinely available clinical variables in the general population remains uncertain. These gaps matter because aortic diseases are uncommon enough that broad imaging-based screening is difficult to implement, but severe enough that improved upstream risk enrichment could have substantial clinical value.

To address these gaps, we performed a comprehensive prospective analysis in the UK Biobank (UKB) and conducted external validation in the China Kadoorie Biobank (CKB). We systematically evaluated the associations of 42 chronic conditions and multimorbidity with incident aortic disease and its subtypes, estimated population attributable fractions (PAFs), and examined sex-specific associations in UKB. We further used genetic analyses to assess the consistency of selected associations and developed prediction models based on routinely collected demographic, clinical, and laboratory variables. Together, these analyses provide large-scale evidence on the chronic disease burden associated with aortic disease and may inform risk stratification and prevention in the general population.

## Results

### Baseline characteristics

Baseline characteristics of the study participants in the UKB and CKB are presented in Tables 1, 2, respectively. The analytical sample comprised 455,945 participants in UKB and 512,721 participants in CKB. During a median follow-up of 14.6 years in UKB and 16.3 years in CKB, 4354 (0.95%) and 5329 (1.04%) participants, respectively, developed aortic disease. Subtype-specific event counts for atherosclerosis of the aorta, aortic dissection, thoracic aortic aneurysm, abdominal aortic aneurysm, and other aneurysms are provided in Supplementary Table 1.

**Table 1:** Baseline characteristics of participants in the UK Biobank

Characteristics	Total	Aortic disease		*P* value
		With	Without	
Number	455,945	4354	451,591	
Age, mean ± SD, years	56.46 ± 8.12	62.11 ± 6.02	56.41 ± 8.11	< 1.00 × 10^−300
BMI, mean ± SD, kg/m^2^	27.30 ± 4.69	28.29 ± 4.49	27.29 ± 4.69	4.92 × 10^−44
Female, n (%)	245,228 (53.78)	1011 (23.22)	244,217 (54.08)	< 1.00 × 10^−300
Race, n (%)				2.89 × 10^−11
White	430,101 (94.33)	4220 (96.92)	425,881 (94.31)	
Black or Black British	7244 (1.59)	38 (0.87)	7206 (1.60)	
Asian or Asian British	10,311 (2.26)	51 (1.17)	10,260 (2.27)	
Mixeda	2698 (0.59)	14 (0.32)	2684 (0.59)	
Other ethnic groupb	5579 (1.22)	31 (0.71)	5548 (1.23)	
Drinking status, n (%)				9.66 × 10^−13
Never	19,257 (4.22)	133 (3.05)	19,124 (4.23)	
Previous	15,532 (3.41)	224 (5.14)	15,308 (3.39)	
Current	420,705 (92.27)	3988 (91.59)	416,717 (92.85)	
Prefer not to answer	439 (0.10)	9 (0.21)	430 (0.10)	
Smoking status, n (%)				4.94 × 10^−295
Never	250,368 (54.91)	1346 (30.91)	249,022 (55.14)	
Previous	157,864 (34.62)	1972 (45.29)	155,892 (34.52)	
Current	46,201 (10.13)	1015 (23.31)	45,186 (10.01)	
Prefer not to answer	1503 (0.33)	21 (0.48)	1482 (0.33)	
Household income, n (%)				8.33 × 10^−101
<£18,000	86,761 (19.08)	1274 (29.50)	85,487 (18.98)	
£18,000-£30,999	100,074 (22.00)	1090 (25.24)	98,984 (21.97)	
£31,000-£51,999	104,163 (22.90)	804 (18.62)	103,359 (22.95)	
£52,000-£100,000	82,579 (18.16)	451 (10.44)	82,128 (18.23)	
>£100,000	22,321 (4.91)	133 (3.08)	22,188 (4.93)	
Missing	58,883 (12.95)	566 (13.11)	58,317 (12.95)	

a Included participants who self-identified as having mixed ethnic background, including White and Black Caribbean, White and Black African, White and Asian, and other mixed backgrounds. b Included Chinese participants, other ethnic groups not otherwise listed, and non-responses (“Do not know” or “Prefer not to answer”). Continuous variables are presented as mean ± standard deviation (SD), and categorical variables are presented as number (*n*) and percentage (%). *P-*values compare participants with and without aortic disease and are two-sided. For continuous variables, one-way analysis of variance was used; for two-group comparisons, this is equivalent to Student’s *t* test. Pearson’s *χ*² tests were used for categorical variables. Abbreviations: *BMI* body mass index; *SD* standard deviation; *£* British pound sterling.

**Table 2:** Baseline characteristics of participants in the China Kadoorie Biobank

Characteristics	Total	Aortic disease		*P* value
		With	Without	
Number	512,721	5329	507,392	
Age, mean ± SD, years	52.03 ± 10.68	58.57 ± 9.14	51.96 ± 10.67	< 1.00 × 10^−300
BMI, mean ± SD, kg/m^2^	23.66 ± 3.38	24.08 ± 3.41	23.65 ± 3.38	8.74 × 10^−20
Female, n (%)	302,518 (59.00)	2888 (54.19)	299,630 (59.05)	8.05 × 10^−13
Region, n (%)				1.32 × 10^−132
Urban	226,190 (44.12)	3235 (60.71)	222,955 (43.94)	
Rural	286,531 (55.88)	2094 (39.29)	284,437 (56.06)	
Drinking status, n (%)				6.80 × 10^−19
Never regular drinker	235,111 (45.86)	2290 (42.97)	232,821 (45.89)	
Ex regular drinker	9256 (1.81)	158 (2.96)	9098 (1.79)	
Occasional or seasonal drinker	163,131 (31.82)	1659 (31.13)	161,472 (31.82)	
Monthly drinker	17,385 (3.39)	170 (3.19)	17,215 (3.39)	
Reduced intake	11,696 (2.28)	188 (3.53)	11,508 (2.27)	
Weekly	76,142 (14.85)	864 (16.21)	75,278 (14.84)	
Smoking status, n (%)				1.04 × 10^−24
Never smoker	317,493 (61.92)	2974 (55.81)	314,519 (61.99)	
Occasional smoker	29,146 (5.68)	315 (5.91)	28,831 (5.68)	
Ex regular smoker	30,561 (5.96)	454 (8.52)	30,107 (5.93)	
Smoker	135,521 (26.43)	1586 (29.76)	133,935 (26.40)	
Household income, n (%)				6.59 × 10^−14
<¥2,500	15,539 (3.03)	154 (2.89)	15,385 (3.03)	
¥2,500-4,999	34,627 (6.75)	300 (5.63)	34,327 (6.77)	
¥5,000-9,999	94,568 (18.44)	822 (15.43)	93,746 (18.48)	
¥10,000-19,999	148,958 (29.05)	1747 (32.78)	147,211 (29.01)	
¥20,000-34,999	126,703 (24.71)	1399 (26.25)	125,304 (24.70)	
≥¥35,000	92,326 (18.01)	907 (17.02)	91,419 (18.02)	

Continuous variables are presented as mean ± standard deviation (SD), and categorical variables are presented as number (*n*) and percentage (%). *P* values compare participants with and without aortic disease and are two-sided. For continuous variables, one-way analysis of variance was used; for two-group comparisons, this is equivalent to Student’s *t* test. Pearson’s *χ*² tests were used for categorical variables. Abbreviations: *BMI* body mass index; *SD* standard deviation; *¥* Chinese yuan.

In both cohorts, participants who developed aortic disease were older than those who did not. In UKB, the mean age was 62.11 ± 6.02 years among participants with aortic disease and 56.41 ± 8.11 years among those without aortic disease; the corresponding values in CKB were 58.57 ± 9.14 years and 51.96 ± 10.67 years, respectively. A similar pattern was observed for body mass index (BMI), with higher mean BMI among participants with aortic disease in both UKB (28.29 ± 4.49 vs. 27.29 ± 4.69 kg/m^2^) and CKB (24.08 ± 3.41 vs. 23.65 ± 3.38 kg/m^2^).

Sex distributions also differed between groups. In UKB, female participants accounted for 53.78% of the overall cohort, but represented only 23.22% of participants with aortic disease, compared with 54.08% among those without aortic disease. In CKB, females constituted 59.00% of the total cohort, 54.19% of the aortic disease group, and 59.05% of the non-aortic disease group. These patterns indicate that aortic disease occurred disproportionately more often in men in both cohorts.

### Associations of chronic conditions with overall and subtype-specific aortic disease incidence

**Fig. 1:**
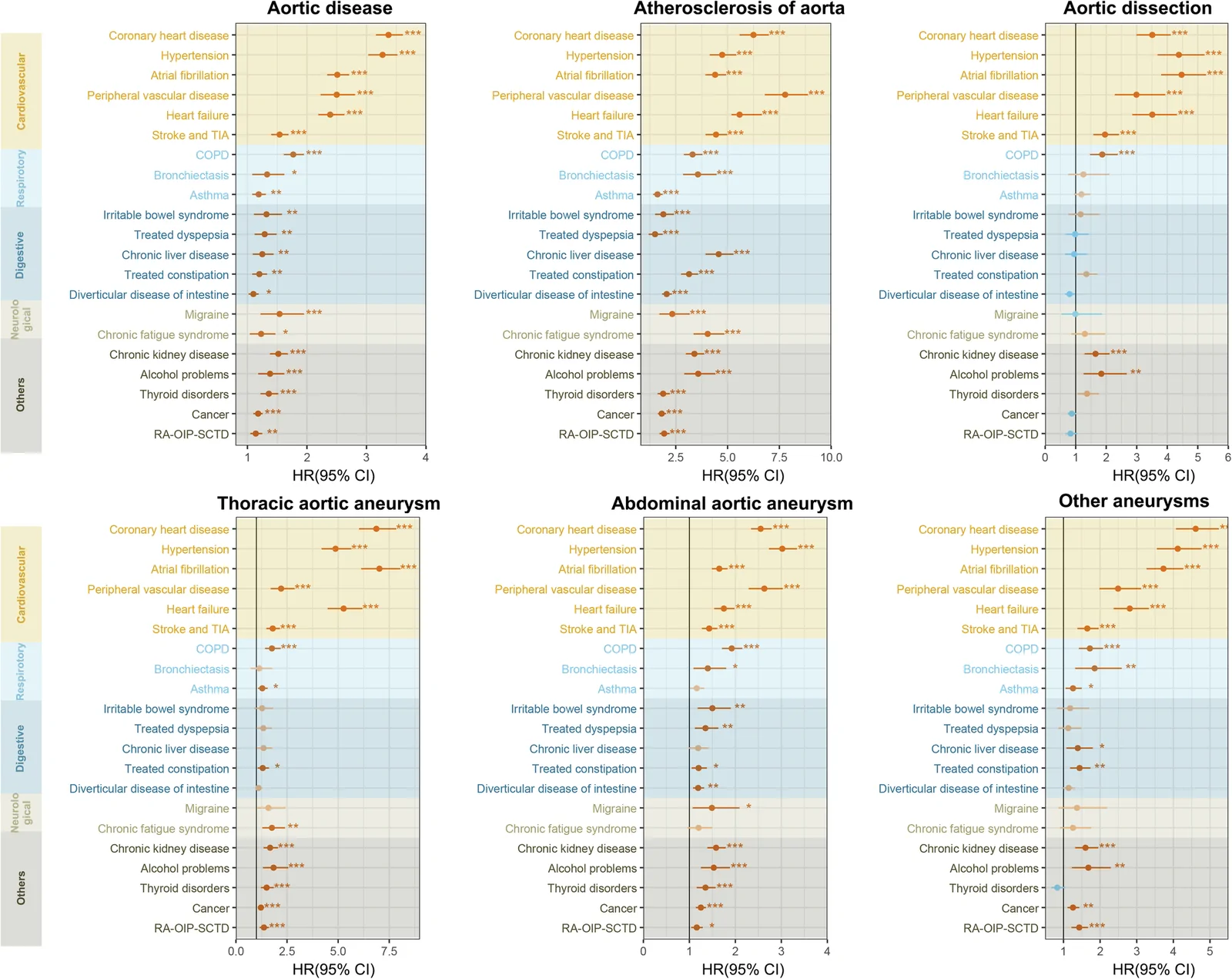
Adjusted hazard ratios for the associations of chronic conditions with overall and subtype-specific aortic disease incidence in UK Biobank. Forest plots show adjusted hazard ratios (HRs) and 95% confidence intervals (CIs) for the associations between baseline chronic conditions and incident overall aortic disease, atherosclerosis of the aorta, aortic dissection, thoracic aortic aneurysm, abdominal aortic aneurysm, and other aneurysms in the UK Biobank. Points indicate adjusted HR estimates, and horizontal whiskers indicate 95% CIs. The UKB analytical sample included *n* = 455,945 participants, among whom *n* = 4354 developed incident aortic disease; endpoint-specific case numbers are provided in Supplementary Table 1. For each chronic condition, participants with the condition were compared with participants without that condition. Models were adjusted for age, sex, assessment centre, ethnicity, smoking status, BMI, drinking status, educational level, household income, and physical activity. *P-*values were calculated using two-sided Wald tests from the Cox proportional hazards models and were adjusted for multiple comparisons using the false discovery rate (FDR) method. Exact nominal and FDR-adjusted *P-*values for each exposure–outcome association are provided in Supplementary Data 1. Symbols indicate FDR-adjusted significance levels: *** *P* < 0.001; ** *P* < 0.01; * *P* < 0.05. Abbreviations: *COPD* chronic obstructive pulmonary disease; *TIA* transient ischaemic attack; *RA-OIP-SCTD* rheumatoid arthritis, other inflammatory polyarthropathies & systematic connective tissue disorders; *HR* hazard ratio; *CI* confidence interval.

Across UKB and CKB, cardiovascular conditions showed the strongest and most consistent associations with incident aortic disease, whereas non-cardiovascular conditions displayed greater heterogeneity across disease subtypes. In UKB, 21 baseline chronic conditions were associated with overall aortic disease after false discovery rate (FDR) correction (Fig. 1 and Supplementary Data 1). The strongest associations were observed for cardiovascular conditions, particularly coronary heart disease (hazard ratios [HR] 3.37, 95% confidence interval [CI] 3.16–3.61), hypertension (3.27, 3.03–3.52), atrial fibrillation (2.51, 2.34–2.71), peripheral vascular disease (2.50, 2.23–2.81), heart failure (2.39, 2.19–2.63), and stroke/transient ischaemic attack (TIA) (1.54, 1.40–1.69). Additional associations were observed for respiratory conditions, including chronic obstructive pulmonary disease (COPD) (1.77, 1.61–1.95), bronchiectasis (1.33, 1.08–1.62), and asthma (1.19, 1.08–1.31). Among digestive conditions, irritable bowel syndrome (1.32, 1.11–1.58), treated dyspepsia (1.29, 1.12–1.49), chronic liver disease (1.25, 1.09–1.44), treated constipation (1.20, 1.08–1.33), and diverticular disease of the intestine (1.10, 1.02–1.19) were associated with increased risk. Among neurological conditions, migraine was positively associated with incident aortic disease (1.54, 1.22–1.95) and chronic fatigue syndrome (1.23, 1.04–1.47). Additional associations were observed for conditions classified as other chronic disorders, including chronic kidney disease (CKD) (1.52, 1.38–1.68), alcohol problems (1.38, 1.18–1.62), thyroid disorders (1.36, 1.22–1.52), cancer (1.18, 1.10–1.26), and rheumatoid arthritis, other inflammatory polyarthropathies & systematic connective tissue disorders (RA-OIP-SCTD) (1.14, 1.05–1.25).

**Fig. 2:**
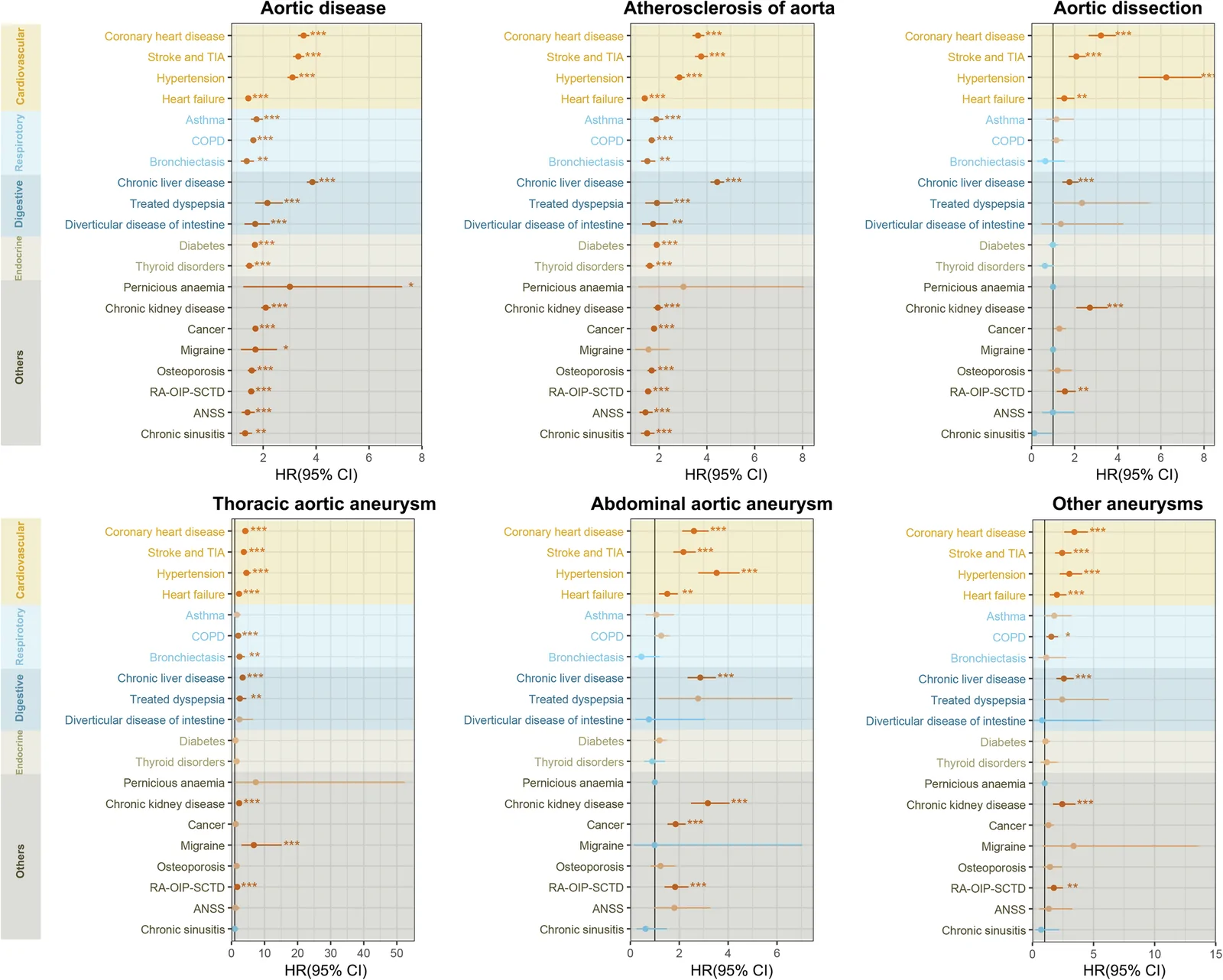
Adjusted hazard ratios for the associations of chronic conditions with overall and subtype-specific aortic disease incidence in China Kadoorie Biobank. Forest plots show adjusted hazard ratios (HRs) and 95% confidence intervals (CIs) for the associations between baseline chronic conditions and incident overall aortic disease, atherosclerosis of aorta, aortic dissection, thoracic aortic aneurysm, abdominal aortic aneurysm, and other aneurysms in the China Kadoorie Biobank. Points indicate adjusted HR estimates, and horizontal whiskers indicate 95% CIs. The CKB analytical sample included *n* = 512,721 participants, among whom *n* = 5329 developed incident aortic disease; endpoint-specific case numbers are provided in Supplementary Table 1. For each chronic condition, participants with the condition were compared with participants without that condition. Models were adjusted for age, sex, region, smoking status, body mass index (BMI), drinking status, educational level, household income, and physical activity. *P-*values were calculated using two-sided Wald tests from the Cox proportional hazards models and were adjusted for multiple comparisons using the false discovery rate (FDR) method. Exact nominal and FDR-adjusted *P-* values for each exposure–outcome association are provided in Supplementary Data 2. Symbols indicate FDR-adjusted significance levels: *** *P* < 0.001; ** *P* < 0.01; * *P* < 0.05. Abbreviations: *COPD* chronic obstructive pulmonary disease; *TIA* transient ischaemic attack; *RA-OIP-SCTD* rheumatoid arthritis, other inflammatory polyarthropathies & systematic connective tissue disorders; *HR* hazard ratio; *CI* confidence interval.

In CKB, the overall pattern was broadly consistent with that observed in UKB (Fig. 2 and Supplementary Data 2). The strongest associations were again seen for cardiovascular conditions, including coronary heart disease (HR 3.53, 95% CI 3.32–3.74), stroke/TIA (3.33, 3.13–3.55), and hypertension (3.11, 2.92–3.32). Positive associations were also observed for respiratory conditions, including asthma (1.75, 1.54–1.99) and COPD (1.63, 1.51–1.74), and for digestive conditions, including chronic liver disease (3.86, 3.65–4.08), treated dyspepsia (2.16, 1.70–2.75) and diverticular disease of the intestine (1.70, 1.29–2.25). Among endocrine conditions, diabetes (1.69, 1.59–1.79) and thyroid disorders (1.48, 1.34–1.64) were positively associated with incident aortic disease. Additional associations were identified for conditions classified as other chronic disorders, including CKD (2.10, 1.93–2.28), cancer (1.71, 1.61–1.82), osteoporosis (1.57, 1.42–1.74), and RA-OIP-SCTD (1.55, 1.43–1.68). Overall, the direction of association was largely consistent across the two cohorts, although the magnitude and breadth of non-cardiovascular associations varied.

Subtype-specific analyses revealed a broadly similar hierarchy of association in UKB and CKB. Aortic atherosclerosis exhibited the broadest comorbidity profile in both cohorts, whereas aortic dissection and the aneurysm subtypes were associated with a narrower range of baseline chronic conditions. Across subtypes, the most consistent associations were observed for hypertension, coronary heart disease, stroke/TIA, CKD, heart failure and COPD, although the magnitude and breadth of non-cardiovascular associations varied between cohorts (Figs. 1, 2 and Supplementary Data 1, 2).

Notably, several non-cardiovascular conditions showed cross-cohort consistency and subtype-level relevance, particularly COPD, CKD, thyroid disorders, and RA-OIP-SCTD, supporting the view that aortic disease risk extends beyond traditional cardiovascular comorbidity. At the same time, some associations appeared to be more cohort-specific. In UKB, conditions such as bronchiectasis, migraine, treated constipation, and irritable bowel syndrome were more prominent, whereas in CKB, diabetes, chronic liver disease, osteoporosis, and cancer showed stronger or broader associations. These differences may indicate population-specific heterogeneity in comorbidity profiles relevant to aortic disease.

### Associations of multimorbidity with overall aortic disease incidence

We next examined whether increasing multimorbidity burden was associated with incident aortic disease in UKB and CKB. In both cohorts, a strong graded association was observed between the number of chronic conditions and the incidence of overall aortic disease. In UKB, compared with participants with 0 chronic conditions, the HRs increased progressively from 2.63 (95% CI 2.18–3.16) for those with 1 condition to 27.18 (23.00–32.11) for those with ≥ 6 conditions. A similar monotonic pattern was observed in CKB, with HRs rising from 4.09 (3.27–5.10) for 1 condition to 54.01 (44.09–66.16) for ≥ 6 conditions. These findings indicate that greater multimorbidity burden was consistently associated with substantially higher incidence of aortic disease across both cohorts (Supplementary Data 3).

### Population-level attributable burden of major chronic conditions in UK Biobank

**Fig. 3:**
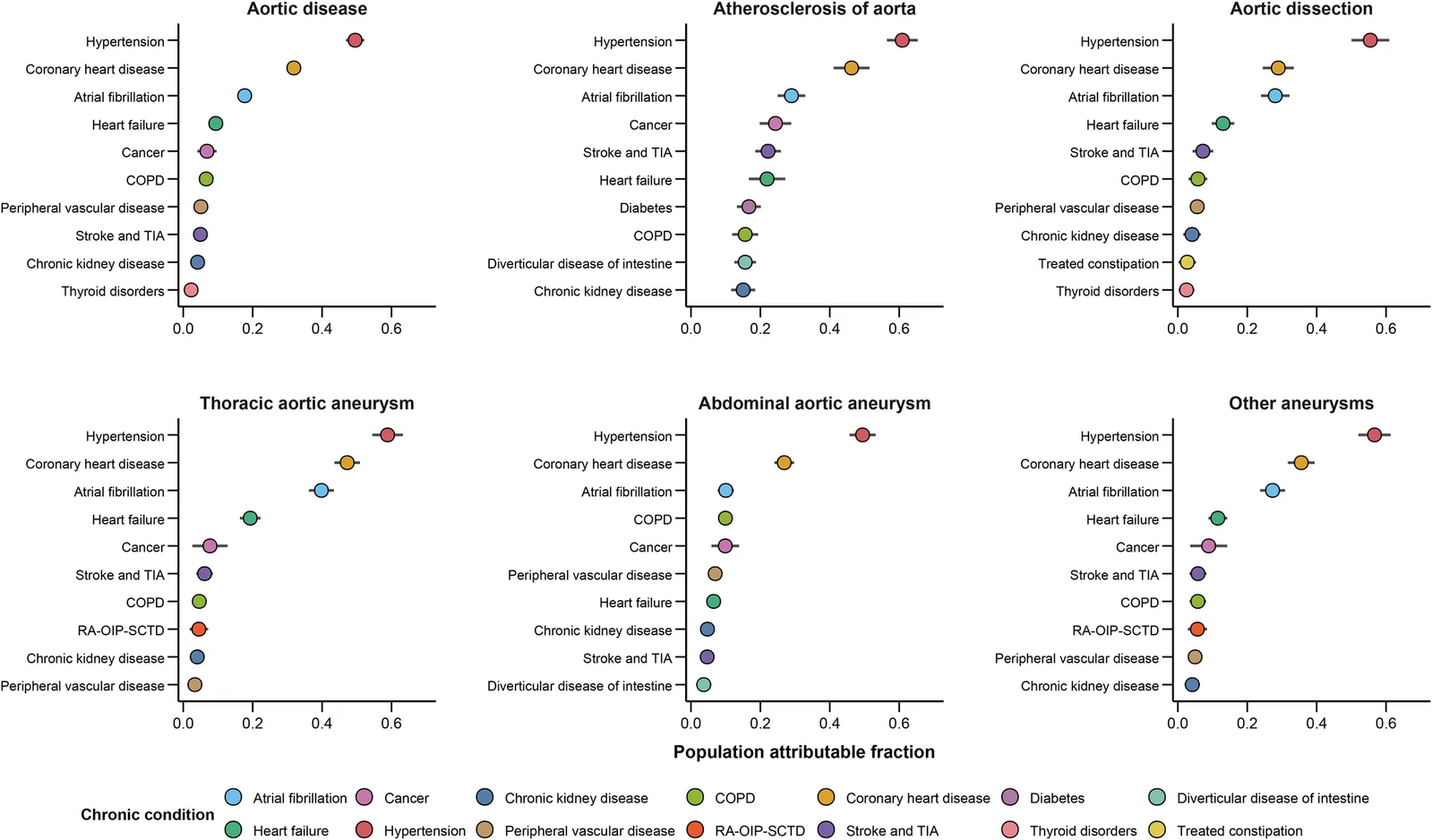
Estimated population attributable fractions for overall and subtype-specific aortic disease in UK Biobank. Dot-and-whisker plots show estimated population attributable fractions (PAFs) and 95% confidence intervals for the top 10 chronic conditions contributing to overall aortic disease, atherosclerosis of the aorta, aortic dissection, thoracic aortic aneurysm, abdominal aortic aneurysm, and other aneurysms in the UK Biobank. The numerical PAF estimates and 95% CIs plotted in this figure are provided in Supplementary Data 4. Points indicate PAF estimates, and whiskers indicate 95% confidence intervals. Colours distinguish chronic conditions. The UKB analytical sample included *n* = 455,945 participants, among whom *n* = 4354 developed incident aortic disease; endpoint-specific case numbers are provided in Supplementary Table 1. PAFs were estimated from multivariable-adjusted Cox models using the same covariates as in the primary UKB association analyses. Adjusted PAF estimates and 95% CIs were obtained using the Greenland–Drescher method with delta method-based variance estimation. Abbreviations: *PAF* population attributable fraction; *COPD* chronic obstructive pulmonary disease; *TIA* transient ischaemic attack; *RA-OIP-SCTD* rheumatoid arthritis, other inflammatory polyarthropathies and systematic connective tissue disorders.

To estimate the population-level burden associated with major chronic conditions, we calculated PAFs in UKB and summarised the top 10 conditions for overall and subtype-specific aortic disease in Fig. 3 and Supplementary Data 4. Among these, hypertension consistently showed the largest attributable fraction across all aortic disease phenotypes. For overall aortic disease, hypertension had the largest estimated population-attributable fraction (49.5%, 95% CI, 46.9–52.1%). The corresponding PAFs were highest for aortic atherosclerosis (60.9%, 95% CI 56.5–65.3%) and thoracic aortic aneurysm (58.9%, 54.5–63.3%), and remained substantial for other aneurysms (56.7%, 52.0–61.3%), aortic dissection (55.5%, 50.0–60.9%), and abdominal aortic aneurysm (49.5%, 45.7–53.2%). Other cardiovascular conditions also contributed materially to the attributable burden. Coronary heart disease showed particularly large PAFs for aortic atherosclerosis (46.3%, 95% CI 41.1–51.4%) and thoracic aortic aneurysm (47.2%, 43.6–50.9%), whereas atrial fibrillation contributed substantially to thoracic aortic aneurysm (39.8%, 36.2–43.4%), aortic atherosclerosis (29.0%, 25.0–32.9%), and other aneurysms (27.3%, 23.7–30.9%).

Several non-cardiovascular conditions also showed measurable attributable fractions, although these were generally smaller. For abdominal aortic aneurysm, the estimated PAFs were 9.9% (95% CI 7.9–12.0%) for COPD and 9.9% (5.9–13.9%) for cancer. CKD had a relatively modest attributable fraction for overall aortic disease (4.1%, 3.0–5.3%), but a larger contribution to aortic atherosclerosis (15.1%, 11.6–18.5%). Overall, these findings indicate that in UKB, hypertension had the largest estimated attributable burden, with additional contributions from coronary heart disease, atrial fibrillation, and selected non-cardiovascular conditions depending on disease subtype.

### Genetic analyses using Mendelian randomisation

To provide genetic support for selected observational associations, we performed Mendelian randomisation (MR) analyses using summary-level data from FinnGen and UKB. Across analyses, genetically predicted hypertension showed the most consistent associations with multiple aortic disease outcomes, including aortic atherosclerosis, aortic dissection, and thoracic and abdominal aortic aneurysm phenotypes (Supplementary Figs. 1–3 and Supplementary Data 5–7). Major coronary heart disease also demonstrated positive associations with several aortic outcomes, particularly aortic atherosclerosis and thoracic aortic aneurysm. Importantly, the genetic signals were not confined to cardiometabolic traits: COPD- and asthma-related phenotypes also showed evidence of association with selected aneurysm outcomes across several analyses, and additional signals were observed for selected non-cardiovascular traits in specific datasets. Additional analyses using different exposure–outcome data sources yielded broadly similar patterns, while also indicating heterogeneity across datasets and disease definitions. Overall, these genetic analyses provide supportive evidence that the observed aortic disease risk profile extends beyond traditional cardiovascular conditions and includes selected non-cardiovascular traits, particularly respiratory phenotypes, although the strength and consistency of these associations varied across outcomes and datasets.

### Machine learning–based prediction of aortic disease

**Fig. 4:**
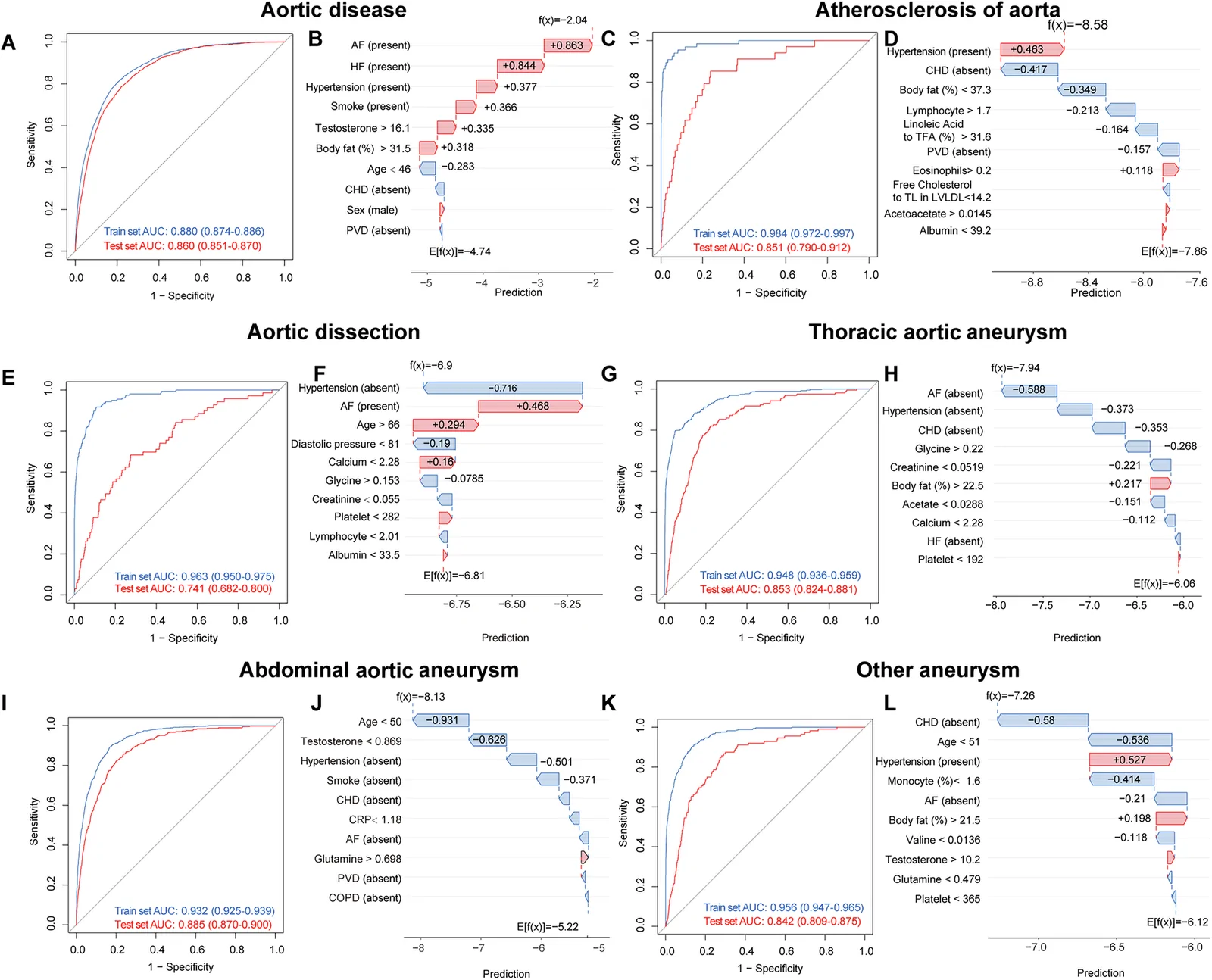
Predictive performance and feature attribution of XGBoost models for aortic disease and its subtypes in UK Biobank. **A**, **C**, **E**, **G**,** I**, **K** Receiver operating characteristic (ROC) curves for XGBoost models predicting overall aortic disease, atherosclerosis of the aorta, aortic dissection, thoracic aortic aneurysm, abdominal aortic aneurysm, and other aneurysms. The red and blue curves represent performance in the test and training datasets, respectively. Areas under the curve (AUCs) with 95% confidence intervals are shown in each panel. **B**, **D**, **F**, **H**, **J**, **L** Shapley additive explanations (SHAP) force plots for representative individuals, showing the top features contributing to each model prediction. For binary variables, labels indicate the presence or absence of the condition. For continuous variables, values are shown together with directional symbols (“>” or “<”) to indicate whether the feature value is above or below the displayed threshold. Features with positive contributions to the predicted risk are shown in red, and those with negative contributions are shown in blue. The length of each bar reflects the magnitude of the feature contribution. Abbreviations: *AF* atrial fibrillation; *CHD* coronary heart disease; COPD, chronic obstructive pulmonary disease; *CRP* C-reactive protein; HF, heart failure; *PVD* peripheral vascular disease; *ROC* receiver operating characteristic; *AUC* area under the curve; *SHAP* Shapley additive explanations.

We next developed XGBoost-based prediction models in UKB for overall and subtype-specific aortic disease using the top 10 predictors ranked by XGBoost gain. For overall aortic disease, the model had an AUROC of 0.880 (95% CI 0.874–0.886) in the training set and 0.860 (0.851–0.870) in the internal test set. Subtype-specific models also performed well, with training-set AUROCs of 0.984 for atherosclerosis of the aorta, 0.963 for aortic dissection, 0.948 for thoracic aortic aneurysm, 0.932 for abdominal aortic aneurysm, and 0.956 for other aneurysms. Corresponding internal test-set AUROCs were 0.851 for atherosclerosis of the aorta, 0.741 for aortic dissection, 0.853 for thoracic aortic aneurysm, 0.885 for abdominal aortic aneurysm, and 0.842 for other aneurysms (Fig. 4).

Shapley additive explanations (SHAP) analyses identified hypertension as the most influential predictor across models, together with age, sex, coronary heart disease, atrial fibrillation, and smoking, while several laboratory markers contributed differentially across subtype-specific models. In the subtype-specific models, albumin and diastolic pressure were prominent for aortic dissection, whereas platelet count, creatinine, C-reactive protein, and several lipid-related measures contributed to discrimination for selected aneurysm phenotypes. Individual-level SHAP force plots further illustrated substantial heterogeneity in feature contributions across representative cases, supporting model interpretability at both the global and individual levels (Supplementary Figs. 4–5).

Overall, machine-learning models integrating chronic conditions with routinely collected variables showed measurable internal discriminatory performance for overall and subtype-specific aortic disease, with interpretable feature attribution. These findings suggest that routinely available clinical information may be useful for future risk stratification of aortic disease; however, independent external validation will be required before any clinical application can be considered.

### Sex-specific associations of chronic conditions with aortic disease in UKB

**Fig. 5:**
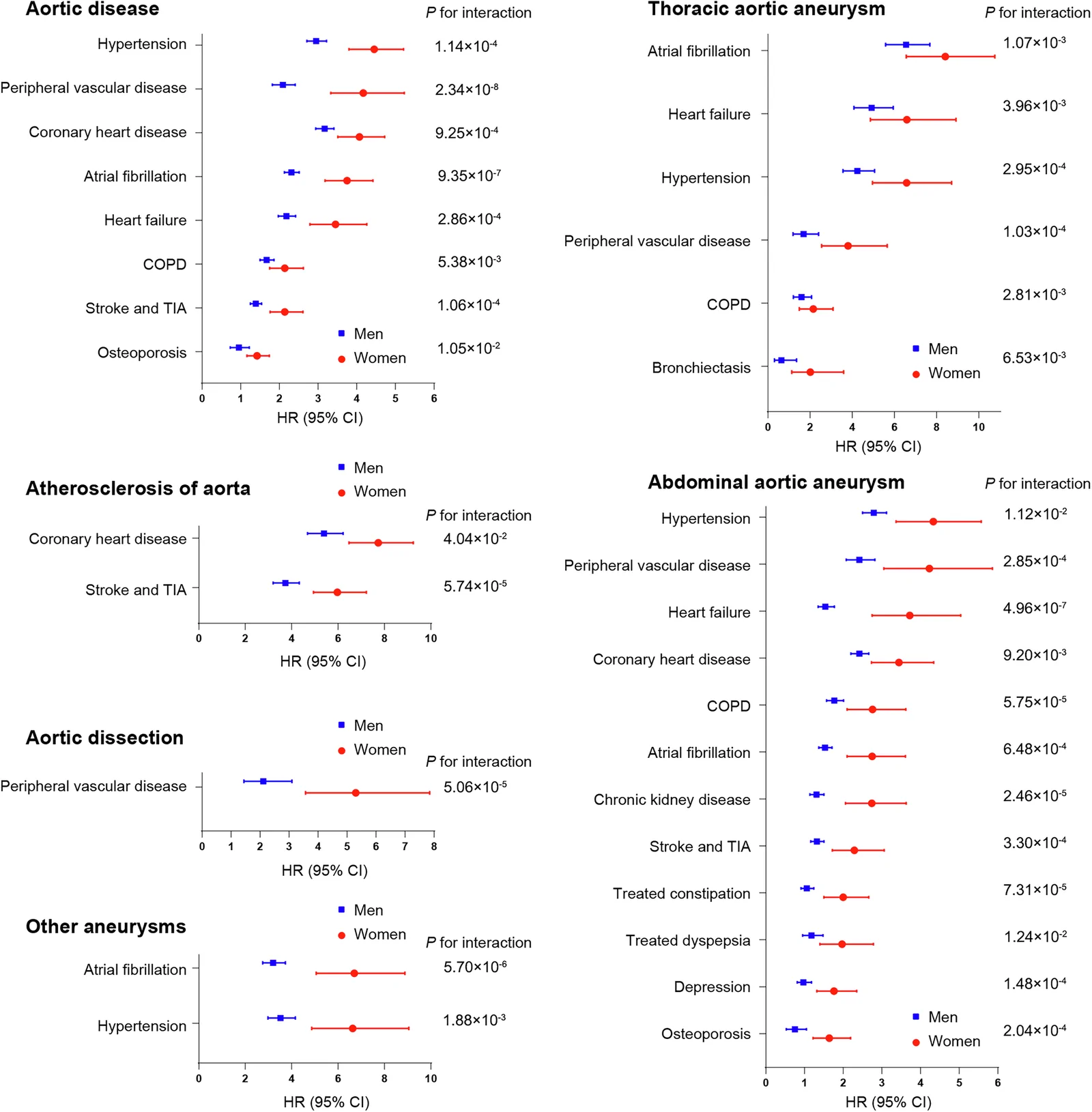
Sex-stratified associations of chronic conditions with aortic disease outcomes in UK Biobank. Forest plots show adjusted hazard ratios (HRs) and 95% confidence intervals (CIs) for associations between selected chronic conditions and incident overall aortic disease, atherosclerosis of the aorta, aortic dissection, thoracic aortic aneurysm, abdominal aortic aneurysm, and other aneurysms, stratified by sex. Points indicate adjusted HR estimates, and horizontal whiskers indicate 95% CIs. Blue squares indicate men, and red circles indicate women. The sex-stratified UKB analytical sample included *n* = 210,717 men and *n* = 245,228 women. For each chronic condition, participants with the condition were compared with participants without that condition within each sex stratum. Models were adjusted for age, assessment centre, ethnicity, smoking status, body mass index, drinking status, educational level, household income, and physical activity. *P-*values for interaction were calculated using two-sided Wald tests for the chronic condition × sex interaction terms in the Cox proportional hazards models. Sex-stratified nominal and FDR-adjusted *P-*values, together with exact interaction *P-*values, are provided in Supplementary Data 8–13. Only conditions with significant sex interactions are displayed. Abbreviations: *COPD* chronic obstructive pulmonary disease; *TIA* transient ischaemic attack; *CKD* chronic kidney disease; *HR* hazard ratio; *CI* confidence interval.

To assess potential effect modification by sex, we performed sex-stratified analyses in UKB and tested interaction terms for the associations between chronic conditions and aortic disease outcomes. Most conditions did not show significant sex differences in relative risk. However, when significant interactions were observed, the associations were generally stronger in female participants than in male participants, despite the higher absolute number of aortic disease events among men across all subtypes (Fig. 5 and Supplementary Data 8–13). Significant interactions for overall aortic disease were observed for hypertension, peripheral vascular disease, coronary heart disease, atrial fibrillation, heart failure, COPD, stroke/TIA, and osteoporosis, with similar patterns across subtype-specific analyses.

### Sensitivity analyses

Sensitivity analyses supported the robustness of the primary findings. Associations remained directionally consistent when accounting for the competing risk of death using cause-specific hazards models (Supplementary Data 14) and when using genetically inferred sex in place of self-reported sex in UKB (Supplementary Data 15).

To address potential outcome misclassification related to administratively coded aortic atherosclerosis, we repeated the main analyses using a clinically specific aortic aneurysm/dissection endpoint restricted to I71.0–I71.9, excluding atherosclerosis of the aorta (I70.0) and inflammatory aortic arch syndrome (M31.4). In UKB, 21 chronic conditions remained associated with the clinically specific endpoint after FDR correction, with the strongest associations observed for coronary heart disease, hypertension, atrial fibrillation, peripheral vascular disease, heart failure, and COPD (Supplementary Data 16). In CKB, 10 chronic conditions remained associated after FDR correction, namely hypertension, coronary heart disease, chronic liver disease, stroke/TIA, chronic kidney disease, cancer, heart failure, RA-OIP-SCTD, COPD, and treated dyspepsia. Although the number of associated conditions was reduced compared with the broad composite endpoint, the retained associations represented a core set of major cardiovascular and systemic conditions, supporting the robustness of the main conclusions.

In a 2-year lag sensitivity analysis excluding aortic disease events occurring within the first 2 years after baseline, the overall pattern of association remained similar (Supplementary Data 17). These findings suggest that the observed associations were unlikely to be fully explained by undiagnosed prevalent aortic disease at baseline.

## Discussion

In this large prospective analysis of the UKB, with external validation in the CKB, we found that both cardiovascular and several non-cardiovascular chronic conditions were associated with incident aortic disease. Among the 42 chronic conditions evaluated, half were associated with increased overall aortic disease risk after FDR correction, with hypertension, coronary heart disease, and atrial fibrillation emerging as the most influential contributors. Importantly, several non-cardiovascular conditions, such as COPD, CKD, and thyroid disorders, were also significantly associated with aortic disease, highlighting the systemic nature of aortic pathology beyond traditional cardiovascular domains. These findings extend previous evidence on lifestyle and classic cardiovascular risk factors by emphasising the potential contribution of multimorbidity, particularly in individuals with pre-existing chronic diseases. MR analyses provided supportive genetic evidence for selected associations between key exposures and aortic disease risk. Moreover, the observed sex-based heterogeneity, with consistently higher relative risks in women, underscores the importance of incorporating sex-specific considerations into risk assessment and prevention strategies.

We found that beyond the well-established cardiovascular contributors, such as hypertension, coronary heart disease, AF, heart failure, and PVD, multiple non-cardiovascular chronic conditions, including COPD, migraine, CKD, thyroid disorders, bronchiectasis, and irritable bowel syndrome, were independently associated with elevated risk of aortic disease. These associations suggest that aortic disease risk may involve systemic processes beyond cardiometabolic factors. COPD and bronchiectasis, for instance, are both characterised by persistent systemic inflammation, dyspnea (chronic hypoxia), and recurrent infections. Inflammation is a common pathological foundation across a wide range of diseases, particularly cardiovascular disorders^10^. Hypoxia was reported to be a critical contributor to both the development and poor prognosis of aortic dissection/aneurysms^11–13^. Mechanistically, hypoxia promotes aortic wall degradation through the ROS-HIF1α-MMPs axis, and clinically, preoperative hypoxemia significantly increases the risk and severity of postoperative acute respiratory distress syndrome, ultimately worsening surgical outcomes in patients with Stanford type A aortic dissection^11,14^. Given the widespread use of fluoroquinolones to manage infections in COPD and bronchiectasis, growing evidence suggests that fluoroquinolones were associated with an increased risk of aortic aneurysm and dissection^15,16^.

Thyroid hormones play a vital role in maintaining cardiovascular health, including the regulation of aortic contractile function. Notably, both low and high levels of T3 were associated with diminished vasoconstrictive responses, forming an inverted U-shaped pattern^17^. In addition, individuals exhibiting low-normal FT4 levels following thoracic endovascular aortic repair face an increased likelihood of aorta-related complications requiring readmission during both short- and intermediate-term follow-up^18^. Irritable bowel syndrome is primarily associated with intestinal infections and gut microbiota dysbiosis, both of which have been linked to an increased risk of aortic dissection/aneurysm^19,20^. Patients with CKD not only exhibit inflammation and reduced renal function, but also experience renal vascular lesions, all of which are closely related to the development and progression of aortic disease^21^. These findings add population-based evidence that chronic conditions beyond the cardiovascular system are associated with aortic disease risk.

Beyond individual conditions, we observed a strong graded association between multimorbidity burden and incident aortic disease. Participants with a greater number of chronic conditions had progressively higher risk, and this pattern was evident in both UKB and CKB. Although the magnitude of some estimates warrants cautious interpretation, particularly for outcomes with lower event counts and for groups with high comorbidity burden, the overall trend suggests that accumulated chronic disease burden may be useful for identifying individuals at particularly high risk. This finding is clinically relevant because it shifts attention from single-condition risk assessment toward a broader, multimorbidity-informed framework for aortic disease surveillance. Although the graded association between multimorbidity burden and aortic disease was evident in both cohorts, the breadth and prominence of specific non-cardiovascular conditions differed between UKB and CKB. These differences may reflect population-specific variation in disease burden, environmental exposures, healthcare systems, and diagnostic practices, and suggest that multimorbidity-informed prevention strategies may need to be adapted to local population contexts. Although some subtype-specific estimates should be interpreted cautiously because of limited event numbers, similar heterogeneity was observed across UKB and CKB, suggesting that the variation may not be due solely to statistical instability but may also reflect differences in the pathophysiology of distinct aortic disease subtypes.

Part of this between-cohort heterogeneity may also reflect the outcome definition used for the broad composite endpoint. Because atherosclerosis of the aorta coded as I70.0 may represent asymptomatic plaque, incidental imaging findings, or previously existing disease detected during routine clinical care, the broader administratively coded composite endpoint should be interpreted with caution. Importantly, when we repeated the analyses using a clinically specific endpoint restricted to aortic dissection and aneurysm diagnoses (I71.0–I71.9), the overall pattern of association remained largely unchanged in UKB and persisted for a core set of conditions in CKB, including hypertension, coronary heart disease, stroke/TIA, chronic kidney disease, chronic liver disease, heart failure, RA-OIP-SCTD, COPD, cancer, and treated dyspepsia. The larger attenuation observed in CKB suggests that the broad composite endpoint in that cohort was more strongly influenced by I70.0 coding. Nevertheless, the persistence of associations for major cardiovascular and systemic conditions supports the robustness of the central conclusion that chronic disease burden is associated with clinically defined aortic dissection and aneurysm outcomes.

The genetic analyses provided supportive evidence for selected observed associations, particularly those involving hypertension and coronary heart disease. Across MR analyses using FinnGen and UKB summary statistics, genetically proxied hypertension showed the most consistent associations with aortic atherosclerosis, dissection, and aneurysm phenotypes, whereas coronary heart disease also showed positive associations with several aortic outcomes. Associations for atrial fibrillation and several non-cardiovascular traits were less consistent across datasets, which may reflect differences in phenotype definition, instrument strength, and genetic heterogeneity. We observed supportive genetic evidence consistent with COPD- and asthma-related traits being relevant to abdominal aortic aneurysm risk, suggesting a possible contribution of chronic pulmonary inflammation in aneurysmal pathogenesis. Taken together, these findings strengthen the plausibility of an aetiologic contribution of major cardiometabolic factors to aortic disease, while also indicating that evidence for several non-cardiovascular traits remains more tentative. This more variable pattern for several non-cardiovascular traits may partly reflect differences in phenotype definition and genetic heterogeneity across datasets. In FinnGen, several clinically defined chronic conditions yielded few genome-wide significant variants under conventional thresholds, and the use of a more pragmatic instrument-selection strategy increased trait coverage but may also have introduced greater uncertainty into some estimates. Accordingly, these MR findings should be interpreted primarily as supportive rather than definitive, particularly for traits showing limited cross-dataset consistency.

The machine learning analyses further suggested that routinely collected clinical and laboratory variables can support risk stratification for aortic disease in the general population. In UKB, XGBoost-based models showed good discrimination for overall aortic disease and variable but generally favourable performance across subtype-specific outcomes. SHAP analyses identified hypertension, age, sex, coronary heart disease, atrial fibrillation, and smoking as among the most influential predictors, whereas selected laboratory markers, including albumin, platelet count, creatinine, C-reactive protein, and lipid-related measures, contributed differentially across phenotypes. These findings suggest shared as well as divergent mechanistic pathways underlying aortic disease phenotypes^22^. However, because these models were developed and internally validated in UKB, further external validation will be required before clinical implementation can be considered.

Stratified analyses showed sex-specific heterogeneity: although men had higher absolute incidence, the associations between chronic conditions and aortic outcomes were generally stronger in female participants among conditions with significant interaction terms^23^. These differences may be driven by biological, clinical, and sociocultural factors. Supporting this, a prior study has demonstrated that, after multivariable adjustment, AF, chronic kidney disease, and a history of coronary artery bypass grafting were associated with significantly higher odds of aortic dissection in female participants than in male participants^24^. Sex hormones are known to significantly contribute to sex differences in cardiovascular disease susceptibility, as reflected by the sharp rise in risk among postmenopausal women. We hypothesise that multiple biological and clinical factors may contribute to this sex-based heterogeneity. Women may experience underdiagnosis or present with atypical symptoms, leading to delayed recognition and less intensive management of chronic cardiovascular conditions, which in turn allows greater cumulative vascular injury before aortic disease manifests^25,26^. In addition, sex differences in arterial remodelling and genetic susceptibility may also play a role, though further research is needed to elucidate these mechanisms. These findings carry important implications for risk stratification and preventive care. While current guidelines for aortic disease primarily emphasise traditional risk factors such as hypertension and family history, our results suggest that a comprehensive assessment of chronic disease burden, particularly in women, may enhance early identification of individuals at elevated risk. Future work should investigate whether multimorbidity-based risk models can improve clinical prediction beyond conventional scores, and whether sex-specific algorithms are warranted.

Several limitations should also be acknowledged. First, despite adjustment for a broad set of covariates, the observational analyses remain susceptible to residual confounding and cannot establish causality. Second, although the inclusion of CKB improved generalisability, the two cohorts differed in ancestry, age structure, disease ascertainment, and healthcare context, which may have contributed to between-cohort heterogeneity. Third, ascertainment of chronic conditions relied on cohort-specific health data sources and may still be subject to misclassification or under-ascertainment, particularly for subclinical or undiagnosed disease. Although participants with any available evidence of prevalent aortic disease at or before baseline were excluded, and a 2-year lag sensitivity analysis was conducted, undiagnosed prevalent aortic disease may still have been misclassified as incident disease. This concern is particularly relevant to atherosclerosis of the aorta coded as I70.0, which may represent asymptomatic, incidental, or pre-existing aortic disease detected during clinical care. Accordingly, findings involving I70.0 were interpreted as exploratory, and additional analyses using a clinically specific aortic dissection and aneurysm endpoint restricted to I71.0–I71.9 were performed to assess robustness. Fourth, some subtype-specific analyses involved relatively small case numbers, which may have reduced precision for selected estimates and contributed to heterogeneity in the corresponding associations. Although some of these subtype-specific patterns were observed across both cohorts, they should still be interpreted cautiously and require further confirmation. Fifth, the MR analyses remain subject to limitations including horizontal pleiotropy, variable instrument strength across traits, and heterogeneity in phenotype definitions across GWAS datasets. Finally, the prediction models were developed and internally validated only in the UKB. The observed train–test performance gaps for some subtype-specific models suggest possible overfitting or limited transportability, and independent external validation is required before clinical application.

In conclusion, this study provides large-scale prospective evidence that a broad spectrum of chronic conditions, extending beyond traditional cardiovascular risk factors, is associated with incident aortic disease. Across UKB, with external validation in CKB, both individual chronic conditions and overall multimorbidity burden were associated with higher aortic disease risk, with the strongest and most consistent associations observed for hypertension, coronary heart disease, and atrial fibrillation, and COPD. Supportive genetic analyses for selected exposures and the performance of prediction models based on routinely collected variables further reinforce the potential value of integrating chronic disease burden into aortic disease risk assessment. Together, these findings suggest that multimorbidity-informed approaches may improve the identification of individuals at elevated risk of aortic disease and support future efforts toward more comprehensive prevention and risk stratification strategies.

## Methods

### Ethics statement

This study complies with all relevant ethical regulations. No new participants were recruited for the present analysis; instead, we used de-identified participant-level data from two established prospective population-based cohorts: the UKB and the CKB. The UKB study has received ethical approval from the NHS North West Multicentre Research Ethics Committee (REC reference. ^16^/NW/0274), and all participants provided written informed consent. This research was conducted using the UKB resource under Application ID 106528. All use of UKB data complied with the UKB data access requirements and the approved research application.

The CKB study (Oxford Tropical Research Ethics Committee approval reference: 025-04) complies with the required ethical standards for medical research involving human participants. Ethical approvals for CKB were granted and have been maintained by the relevant institutional ethical research committees in the UK and China, including the Oxford Tropical Research Ethics Committee, the Ethical Review Committee of the Chinese Centre for Disease Control and Prevention, the Chinese Academy of Medical Sciences, and the Institutional Review Board of Peking University. All CKB participants provided written informed consent at each survey visit, including consent for access to medical records and long-term storage of biospecimens for future medical research. The present study used data from the CKB resource and complied with CKB data access requirements, institutional approvals, and data-use conditions.

Information on participant compensation in the original cohorts was not available to the authors from the data providers for the present analysis.

### Study population

This study included participants from two large prospective population-based cohorts: the UKB and the CKB. UKB recruited more than 500,000 adults aged 37–73 years between 2006 and 2010 from 22 assessment centres across England, Scotland, and Wales. At baseline, participants completed touchscreen and nurse-led questionnaires, underwent physical measurements, and provided blood samples for genetic and biomarker assays. Longitudinal follow-up in UKB is conducted through linkage to electronic health records, including hospital inpatient data (ICD-10), death registries and primary care data.

CKB recruited more than 512,000 adults aged 30–79 years between 2004 and 2008 from 10 geographically diverse areas of China (five urban and five rural). At local assessment centres, trained health workers administered a laptop-based questionnaire, performed physical measurements, and collected blood samples for long-term storage and on-site testing. Follow-up for disease outcomes in CKB was achieved through linkage to death and disease registries and to health insurance records, with events centrally processed and coded according to the ICD-10.

For each of the 42 chronic conditions, we excluded participants with missing covariate data, those lost to follow-up, those with evidence of prevalent aortic disease at or before baseline, and those whose recorded onset of aortic disease preceded the corresponding chronic condition. To establish the absence of aortic disease at baseline, prevalent aortic disease was defined as any record of aortic disease before or at the baseline assessment in the available cohort-specific data sources. In UKB, these sources included hospital inpatient diagnosis records (Fields 41270 and 41280), aortic-related operative procedure records (Fields 41272 and 41282); primary care records and self-reported medical history were additionally considered where available and if included in the analytic pipeline. In CKB, prevalent aortic disease was identified using baseline survey information and linked electronic health records, including disease registries, and health insurance-based hospital records. Participants with any pre-baseline record of atherosclerosis of the aorta (I70.0), aortic dissection and aneurysm (I71.0–I71.9), inflammatory aortic arch syndrome (M31.4), or corresponding aortic procedure codes were excluded from the relevant analyses.

### Assessment of chronic conditions

Chronic conditions were defined using cohort-specific health data sources and harmonised into a common analytical framework. In the UKB, chronic conditions were ascertained from linked ICD-10 diagnosis records (Field 41270), derived from hospital inpatient data. In the CKB, chronic conditions were ascertained using available baseline survey data and linked electronic health records, including disease registries and health insurance records, and were harmonised to the same disease categories as those used in UKB wherever possible^27^. A total of 42 chronic conditions were selected based on clinical relevance and harmonised across cohorts using the disease definitions^28–30^. Conditions with insufficient case numbers for stable estimation, including hearing loss, dementia, blindness, and learning disabilities, were excluded from the present analysis. In CKB, polycystic ovary syndrome was not included in the analyses because fewer than three exposed participants were identified. The corresponding disease definitions and ICD-10 codes are provided in Supplementary Data 18. Multimorbidity was defined as the coexistence of two or more chronic conditions in the same individual.

### Ascertainment of aortic disease

Incident aortic disease was ascertained in each cohort using linked health record data. In UKB, case identification was based on national death registries (Fields 40001 and 40002), hospital inpatient diagnoses (Fields 41270 and 41280), and aortic-related surgical procedure records (Fields 41272 and 41282; procedure codes listed in Supplementary Data 19). In CKB, aortic disease outcomes were identified through linkage to death registries, disease registries, and health insurance-based hospital records, with events centrally processed and coded using ICD-10.

In the main composite analysis, aortic disease was defined using ICD-10 codes and included atherosclerosis of aorta (I70.0), aortic dissection (I71.0), thoracic aortic aneurysm with or without rupture (I71.1 and I71.2), abdominal aortic aneurysm with or without rupture (I71.3 and I71.4), other aortic aneurysms (I71.5, I71.6, I71.8, and I71.9), and inflammatory aortic arch syndrome (M31.4). Because M31.4 was rare, it was included in the overall aortic disease outcome but not analysed separately in subtype-specific analyses.

Participants were followed from baseline until the first occurrence of incident aortic disease, death, or the end of follow-up, whichever came first.

### Covariates

Covariates were selected a priori as potential confounders based on epidemiological and clinical relevance^1,24^. In both cohorts, adjustment variables included age, sex, smoking status, body mass index, drinking status, educational level, household income, and physical activity, together with a cohort-specific geographic variable (UKB assessment centre in UKB and region in CKB).

In UKB, age was modelled in years, sex was categorised as female or male, and the assessment centre was included as a geographic covariate. Ethnicity was additionally adjusted for and classified as White, Black or Black British, Asian or Asian British, Mixed heritage, or Other ethnic groups. Educational attainment was categorised into seven groups: college/university degree, Advanced/Advanced Subsidiary Levels or equivalent, Ordinary Levels/General Certificate of Secondary Education or equivalent, Certificate of Secondary Education or equivalent, National Vocational Qualification/Higher National Diploma/Certificate or equivalent, other professional qualifications, and no formal qualifications. Annual household income was grouped into five categories: <£18,000, £18,000–£30,999, £31,000–£51,999, £52,000–£100,000, and >£100,000. Smoking and drinking status were classified as never, previous, or current.

In CKB, age was modelled in years, sex was categorised as female or male, and region was classified according to the 10 recruitment areas, comprising five urban regions (Qingdao, Harbin, Haikou, Suzhou, and Liuzhou) and five rural regions (Sichuan, Gansu, Henan, Zhejiang, and Hunan). Educational level was categorised as no formal school, primary school, middle school, high school, technical school/college, and university. Annual household income was grouped into six categories: <¥2,500, ¥2,500–4,999, ¥5,000–9,999, ¥10,000–19,999, ¥20,000–34,999, and ≥¥35,000. Smoking status was classified as never smoker, occasional smoker, ex-regular smoker, or current smoker, and drinking status as never regular drinker, ex-regular drinker, occasional or seasonal drinker, monthly drinker, reduced intake, or weekly drinker.

In both cohorts, BMI was calculated as weight in kilograms divided by height in metres squared (kg/m^2^). Physical activity was quantified using metabolic equivalent of task (MET) values. Sex was based on self-report at baseline in the primary analyses in both cohorts. Sex was considered in the study design and statistical analyses: primary models were adjusted for sex, and sex-stratified analyses with chronic condition × sex interaction terms were performed in UKB. Gender identity was not analysed separately because harmonised gender identity information was not available in the analytical datasets.

### Statistical analysis

Continuous variables are presented as mean ± standard deviation (SD) and were compared using one-way analysis of variance; for two-group comparisons, this is equivalent to Student’s *t* test. Categorical variables are presented as counts (percentages) and were compared using Pearson’s *χ*² tests.

### Association analyses of chronic conditions and multimorbidity with aortic disease

Associations of baseline chronic conditions with incident aortic disease were evaluated separately in UKB and CKB using Cox proportional hazards regression models. Participants were followed from baseline until the first occurrence of incident aortic disease, death, or the end of follow-up, whichever came first. HR and 95% CI were estimated for each of the 42 chronic conditions.

In UKB, models were adjusted for age, sex, assessment centre, ethnicity, smoking status, BMI, drinking status, educational level, household income, and physical activity. In CKB, models were adjusted for age, sex, region, smoking status, BMI, drinking status, educational level, household income, and physical activity.

Multicollinearity among the adjusted covariates was evaluated using variance inflation factors, and all covariates showed variance inflation factors < 2, indicating no evidence of problematic collinearity. To account for multiple testing, *P-*values were adjusted using the FDR correction method.

Multimorbidity analyses were conducted by modelling the number of chronic conditions as a categorical exposure, using participants with 0 chronic conditions as the reference group. Tests for trend were performed by modelling the number of chronic conditions as a continuous variable.

### Population attributable fraction analyses

To estimate the population-level burden associated with major chronic conditions, PAFs were calculated in the UKB only. PAFs were estimated for the leading associated conditions using the Greenland–Drescher method, which extends the Cox proportional hazards model to derive adjusted attributable fractions in cohort studies. The same covariates as in the primary UKB Cox models were included to ensure consistency. Adjusted point estimates and 95% CIs were obtained using delta method-based variance estimation.

### Mendelian randomisation analysis

To assess whether selected associations between chronic conditions and aortic disease were supported by genetic evidence, we performed MR analyses using summary-level genome-wide association study (GWAS) data from FinnGen and UK Biobank-derived GWAS resources^31,32^. Genetic instruments for each chronic condition were selected from the corresponding GWAS summary statistics. In UKB, we used the conventional genome-wide significance threshold (*P* < 5 × 10^−8^) and retained variants with *F*-statistic > 10 as instrumental variables. In FinnGen, because several disease traits yielded only a limited number of eligible variants under the conventional threshold, likely reflecting phenotypic and genetic heterogeneity across clinically defined disease categories, a pragmatic thresholding strategy was applied. The initial selection threshold was set at *P* < 5 × 10^−6^, with exclusion of weak instruments (*F*-statistic < 10). When fewer than five eligible variants were available under this threshold, the significance threshold was further relaxed to *P* < 5 × 10^−5^, while requiring *F*-statistic ≥ 15 to retain instrument strength. Independent variants were identified by linkage disequilibrium clumping (*r*² < 0.001, 10,000 kb window).

Outcome datasets included ICD-coded diagnoses of atherosclerosis of the aorta, aortic dissection, and aortic aneurysm, including thoracic and abdominal subtypes. Exposure and outcome datasets were harmonised to align effect alleles and minimise strand ambiguity. The inverse-variance weighted (IVW) method was used as the primary MR approach. MR-Egger, weighted median, simple mode, and weighted mode analyses were performed as sensitivity analyses to evaluate the robustness of the findings under different assumptions regarding pleiotropy and instrument validity. When estimates were broadly consistent in direction across methods, the IVW results were emphasised. When substantial discordance was observed, the results were interpreted cautiously.

MR analyses were first performed within each available data source to evaluate genetically proxied associations between chronic conditions and aortic disease outcomes. To reduce potential bias due to sample overlap, additional cross-dataset MR analyses were conducted using exposure and outcome summary statistics derived from different sources.

### Machine learning model establishment and feature selection

Machine learning models were developed in UKB to predict overall and subtype-specific aortic disease using the XGBoost algorithm. Candidate predictors included 329 baseline demographic, clinical, chronic disease, and laboratory variables routinely available in UKB (Supplementary Data 20). Participants with complete data for the selected variables were randomly divided into a training set (70%) and a test set (30%). Within the training set, features were ranked according to XGBoost gain, and the top 10 predictors for each outcome were retained to construct parsimonious final models. Final XGBoost models were then trained in the training set and evaluated in the held-out test set. Model discrimination was assessed using the area under the receiver operating characteristic curve (ROC-AUC). To improve interpretability, we applied Shapley Additive exPlanations (SHAP) to quantify the contribution of each predictor to model output at both the global level (overall feature importance across participants) and the individual level (feature contributions for representative predictions). Global importance was summarised by aggregating the absolute SHAP values across individuals, and local explanations were visualised using SHAP force plots for representative participants^33–35^.

### Stratified and sensitivity analyses

Sex-stratified analyses were performed in UKB to assess potential effect modification by sex. Multiplicative interaction terms between sex and each chronic condition were included in the corresponding Cox proportional hazards models, and statistical significance was defined as *P* for interaction < 0.05.

Sensitivity analyses were conducted to assess the robustness of the findings. First, to account for the competing risk of death, cause-specific hazards models were fitted with incident aortic disease as the event of interest and all-cause mortality as the competing event. These analyses were implemented using the CSC function in the *riskRegression* package in R. Results from these models are reported as cause-specific hazard ratios (csHRs) with 95% confidence intervals. Second, because a small number of UKB participants showed discordance between self-reported sex and genetically inferred sex, we repeated the analyses using genetically inferred sex in place of self-reported sex to assess the potential impact of sex misclassification. Third, because atherosclerosis of the aorta coded as I70.0 may capture asymptomatic, incidental, or previously existing aortic atherosclerosis detected during clinical care, findings involving I70.0 were interpreted cautiously and considered exploratory. We therefore performed an additional sensitivity analysis using a clinically specific aortic dissection and aneurysm endpoint restricted to I71.0–I71.9, excluding atherosclerosis of the aorta (I70.0) and inflammatory aortic arch syndrome (M31.4). Fourth, to further minimise the possibility that undiagnosed prevalent aortic disease was misclassified as incident disease, we performed a 2-year lag sensitivity analysis by excluding aortic disease events occurring within the first 2 years after baseline.

All statistical analyses were conducted in R version 4.5.1 (R Foundation for Statistical Computing, Vienna, Austria). Cox proportional hazards models were fitted using the survival package version 3.6.4. Competing-risk analyses were performed using the riskRegression package version 2025.9.17. Mendelian randomisation analyses were conducted using TwoSampleMR version 0.7.0 and ieugwasr version 1.1.0. Machine-learning analyses were performed using xgboost version 1.7.11.1. Model interpretability was assessed using SHAP values computed with the built-in Tree SHAP implementation in xgboost and visualised using shapviz version 0.10.3. Data visualisation was performed using ggplot2 version 3.5.1. A two-sided *P* < 0.05 was considered statistically significant, with FDR correction applied where multiple testing was involved.

### Reporting summary

Further information on research design is available in the Nature Portfolio Reporting Summary linked to this article.

## Supplementary information

Supplementary Information

Description of Additional Supplementary Files

Supplementary Data 1-20

Reporting Summary

Transparent Peer Review file

## Data Availability

The summary-level data generated in this study are provided as Supplementary Data file 1–20 accompanying this article and constitute the minimum dataset necessary to interpret, verify, and extend the findings. These data do not contain identifiable participant-level information. The individual-level UK Biobank data used in this study are available under restricted access because they contain participant-level health, genetic, biomarker, and linked electronic health-record information governed by participant consent, data protection requirements, and UK Biobank data access policies. These data cannot be publicly deposited or redistributed by the authors. This study used UK Biobank data under Application ID 106528. Access can be obtained by bona fide researchers for approved health-related research through the UK Biobank Access Management System, subject to UK Biobank review and completion of relevant data access agreements. The response timeframe and duration of data availability after approval are determined by UK Biobank. Individual-level China Kadoorie Biobank data are also available under restricted access and cannot be publicly deposited or redistributed by the authors. Access may be requested by qualified researchers for scientifically and ethically approved research through the China Kadoorie Biobank data access procedure [https://www.ckbiobank.org/data-access], subject to review and project-specific authorisation by the relevant CKB data access committee and participating institutions. The authors are not permitted to redistribute CKB individual-level data. The expected response timeframe is up to 16 weeks in total, including formal application, review of proposal, approval, and signing of the Data Access Agreement, and data are available after approval for the period specified in the approved data access agreement or project authorisation. GWAS summary statistics used in this study are publicly available from FinnGen [https://r12.finngen.fi] and UK Biobank [https://pan.ukbb.broadinstitute.org].

## Code availability

The code used for data cleaning, statistical analyses, Mendelian randomisation analyses, and machine learning modelling in this study is publicly available on GitHub at https://github.com/zhugecaoming/ukb-ckb-aortic-disease-code and has been archived in Zenodo as a published, version-specific software record. https://doi.org/10.5281/zenodo.21620343.
