# Supplementary material for: Chronic conditions and aortic disease risk: a prospective cohort study with predictive and etiological analyses
Source: Nat Commun. 2026 Aug 11;17:9662. doi: 10.1038/s41467-026-76551-y (PMC13558640; doi:10.1038/s41467-026-76551-y)
Supplement: Supplementary file 1 [file 41467_2026_76551_MOESM1_ESM.pdf]

Chronic Conditions and Aortic Disease Risk: A Prospective Cohort Study with Predictive and Etiological Analyses

Supplementary Table 1:

Supplementary Table 1 | Numbers of overall and subtype-specific aortic disease events in the UK Biobank and China Kadoorie Biobank.

Supplementary Data 1–20 (provided as Excel file):

Supplementary Data 1 | Associations of chronic conditions with overall and subtype-specific incident aortic diseases: Cox proportional hazards models in the UK Biobank

Supplementary Data 2 | Associations of chronic conditions with overall and subtype-specific incident aortic diseases: Cox proportional hazards models in the China Kadoorie Biobank

Supplementary Data 3 | Association of the number of chronic conditions with overall aortic disease incidence

Supplementary Data 4 | Estimated population attributable fractions for overall and subtype-specific aortic disease in UK Biobank

Supplementary Data 5 | Mendelian randomization analyses of genetically proxied chronic conditions and aortic disease outcomes in FinnGen

Supplementary Data 6 | Cross-dataset Mendelian randomization analyses of genetically proxied chronic conditions in FinnGen and aortic disease outcomes in UK Biobank

Supplementary Data 7 | Mendelian randomization analyses of genetically proxied chronic conditions and aortic disease outcomes in UK Biobank

Supplementary Data 8 | Sex-specific associations of chronic conditions with incident aortic disease in UK Biobank

Supplementary Data 9 | Sex-specific associations of chronic conditions with incident aortic atherosclerosis in UK Biobank

Supplementary Data 10 | Sex-specific associations of chronic conditions with incident aortic dissection in UK Biobank

Supplementary Data 11 | Sex-specific associations of chronic conditions with incident thoracic aortic aneurysm in UK Biobank

Supplementary Data 12 | Sex-specific associations of chronic conditions with incident abdominal aortic aneurysm in UK Biobank

Supplementary Data 13 | Sex-specific associations of chronic conditions with incident other aneurysms in UK Biobank

Supplementary Data 14 | Adjusted cause-specific hazard ratios for the association between chronic conditions and incident aortic disease in UK Biobank: competing-risk analysis

Supplementary Data 15 | Sensitivity analysis of the association between chronic conditions and incident aortic disease in UK Biobank using genetically inferred sex

Supplementary Data 16 | Sensitivity analysis of the association between chronic conditions and incident aortic dissection and aortic aneurysm in UK Biobank and China Kadoorie Biobank

Supplementary Data 17 | Sensitivity analysis of the associations between chronic conditions and incident aortic disease after excluding events occurring within the first 2 years of follow-up in UK Biobank and China Kadoorie Biobank

Supplementary Data 18 | Disease definitions, ICD-10 codes, and cohort-specific sample sizes for the 42 chronic conditions

Supplementary Data 19 | Definition of surgery for aortic disease in the UK Biobank

Supplementary Data 20 | Features incorporated into the XGBoost predictive model (n=329)

Supplementary Figures 1–5:

Supplementary Figure 1 | Mendelian randomization analyses of genetically proxied chronic conditions and aortic disease outcomes in FinnGen.

Supplementary Figure 2 | Cross-dataset Mendelian randomization analyses of genetically proxied chronic conditions in FinnGen and aortic disease outcomes in UK Biobank.

Supplementary Figure 3 | Mendelian randomization analyses of genetically proxied chronic conditions and aortic disease outcomes in UK Biobank.

Supplementary Figure 4 | SHAP force plots for representative individual predictions from XGBoost models in the UK Biobank.

Supplementary Figure 5 | Beeswarm plots of SHAP values for the top 10 features in the XGBoost models.

Supplementary Table 1. Numbers of overall and subtype-specific aortic disease events in the UK Biobank and China Kadoorie Biobank

**Outcome**	**UKB, *n***	**CKB, *n***
Overall aortic disease	4,354	5,329
Atherosclerosis of aorta	1,423	4,075
Aortic dissection	792	501
Thoracic aortic aneurysm	1,133	387
Abdominal aortic aneurysm	2,392	436
Other aneurysms	1,184	249

Note: Because participants could contribute to more than one subtype category, subtype-specific event counts do not necessarily sum to the total number of overall aortic disease cases.

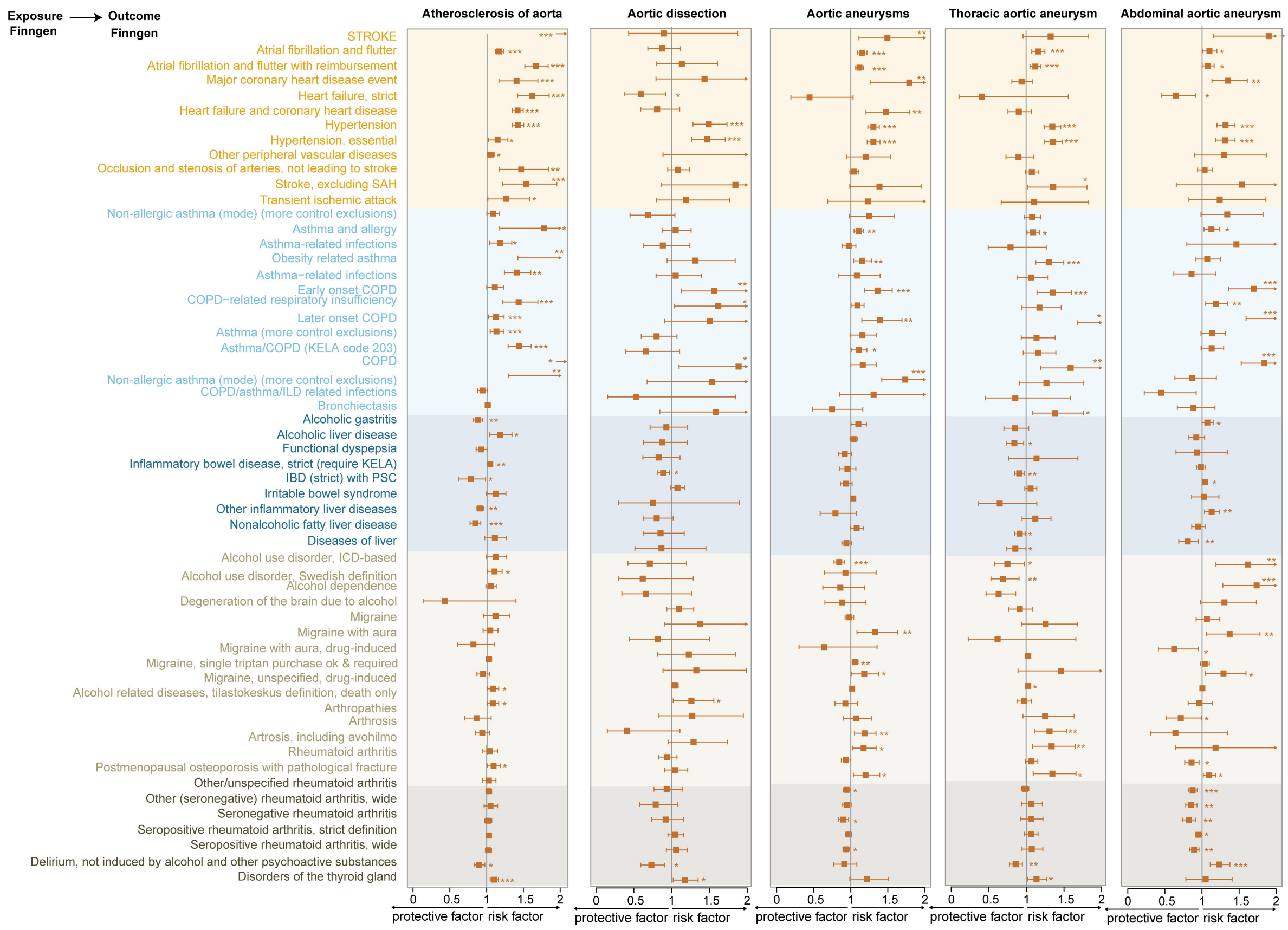

Supplementary Figure 1. Mendelian randomization analyses of genetically proxied chronic conditions and aortic disease outcomes in FinnGen.

Forest plots show odds ratios (ORs) and 95% confidence intervals (CIs) from Mendelian randomization (MR) analyses using FinnGen summary statistics for both exposures and outcomes. Outcomes include aortic atherosclerosis, aortic dissection, aortic aneurysm, thoracic aortic aneurysm and abdominal aortic aneurysm. Points indicate OR estimates, and horizontal whiskers indicate 95% CIs. The vertical dashed line indicates the null value (OR = 1). *P* values were two-sided and were adjusted for multiple comparisons using the false discovery rate (FDR) method, where applicable. Exact *P* values and FDR-adjusted *P* values are provided in the corresponding Supplementary Data 5. Symbols indicate FDR-adjusted significance levels for visual reference: ***$P < 0.001$; **$P < 0.01$; *$P < 0.05$. Background shading indicates broad categories of exposure traits. No bar charts, representative blots, micrographs or gel images are shown. Abbreviations: OR, odds ratio; CI, confidence interval; COPD, chronic obstructive pulmonary disease; SAH, subarachnoid haemorrhage; IBD, inflammatory bowel disease.

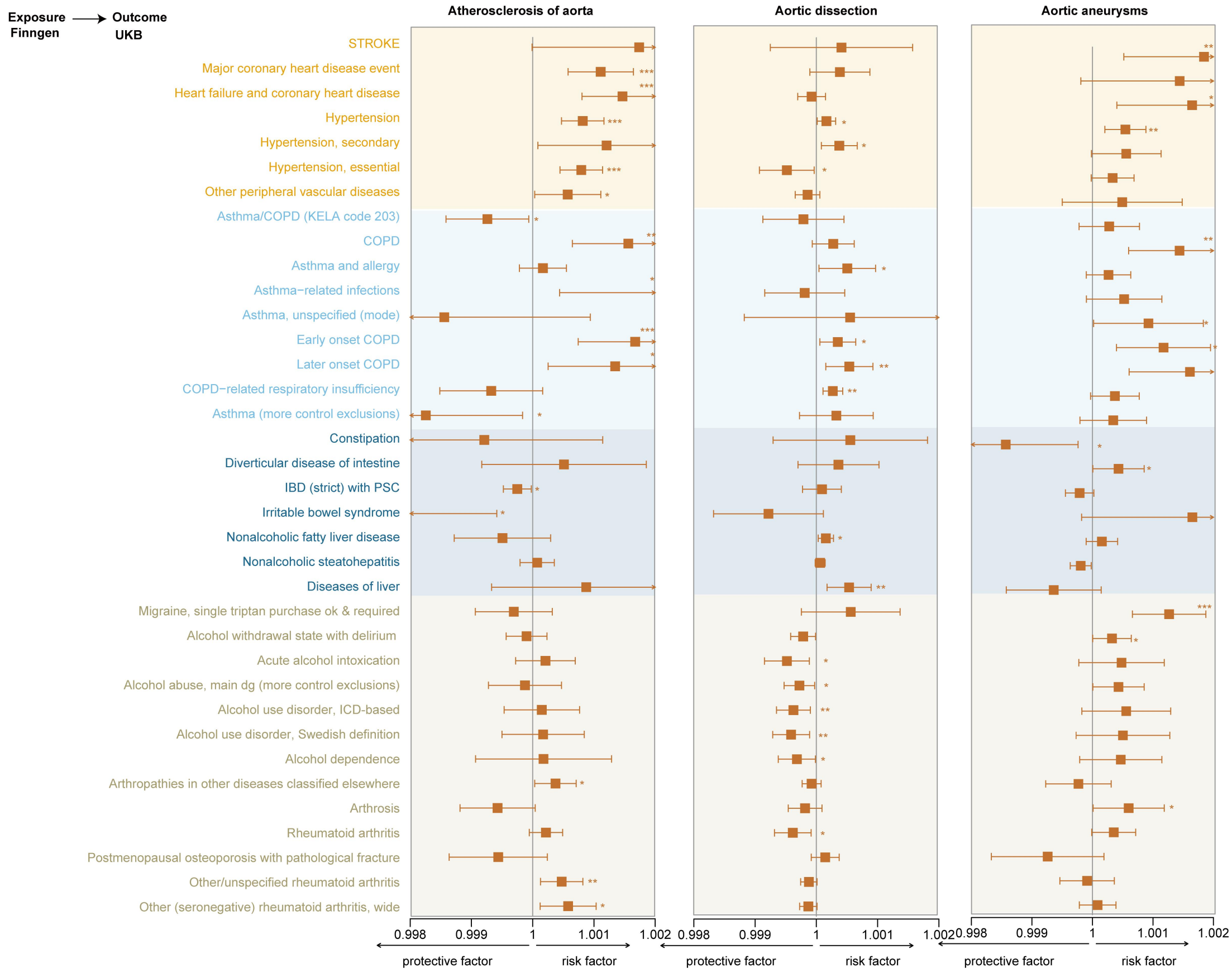

Supplementary Figure 2. Cross-dataset Mendelian randomization analyses of genetically proxied chronic conditions in FinnGen and aortic disease outcomes in UK Biobank.

Forest plots show ORs and 95% CIs from cross-dataset MR analyses using FinnGen summary statistics for exposures and UK Biobank summary statistics for outcomes. Outcomes include aortic atherosclerosis, aortic dissection and aortic aneurysm. Points indicate OR estimates, and horizontal whiskers indicate 95% CIs. The vertical dashed line indicates the null value (OR = 1). *P* values were two-sided and were adjusted for multiple comparisons using the FDR method, where applicable. Exact *P* values and FDR-adjusted *P* values are provided in the corresponding Supplementary Data 6. Symbols indicate FDR-adjusted significance levels for visual reference: ***$P < 0.001$; **$P < 0.01$; *$P < 0.05$. Background shading indicates broad categories of exposure traits. No bar charts, representative blots, micrographs or gel images are shown. Abbreviations: OR, odds ratio; CI, confidence interval; COPD, chronic obstructive pulmonary disease; IBD, inflammatory bowel disease.

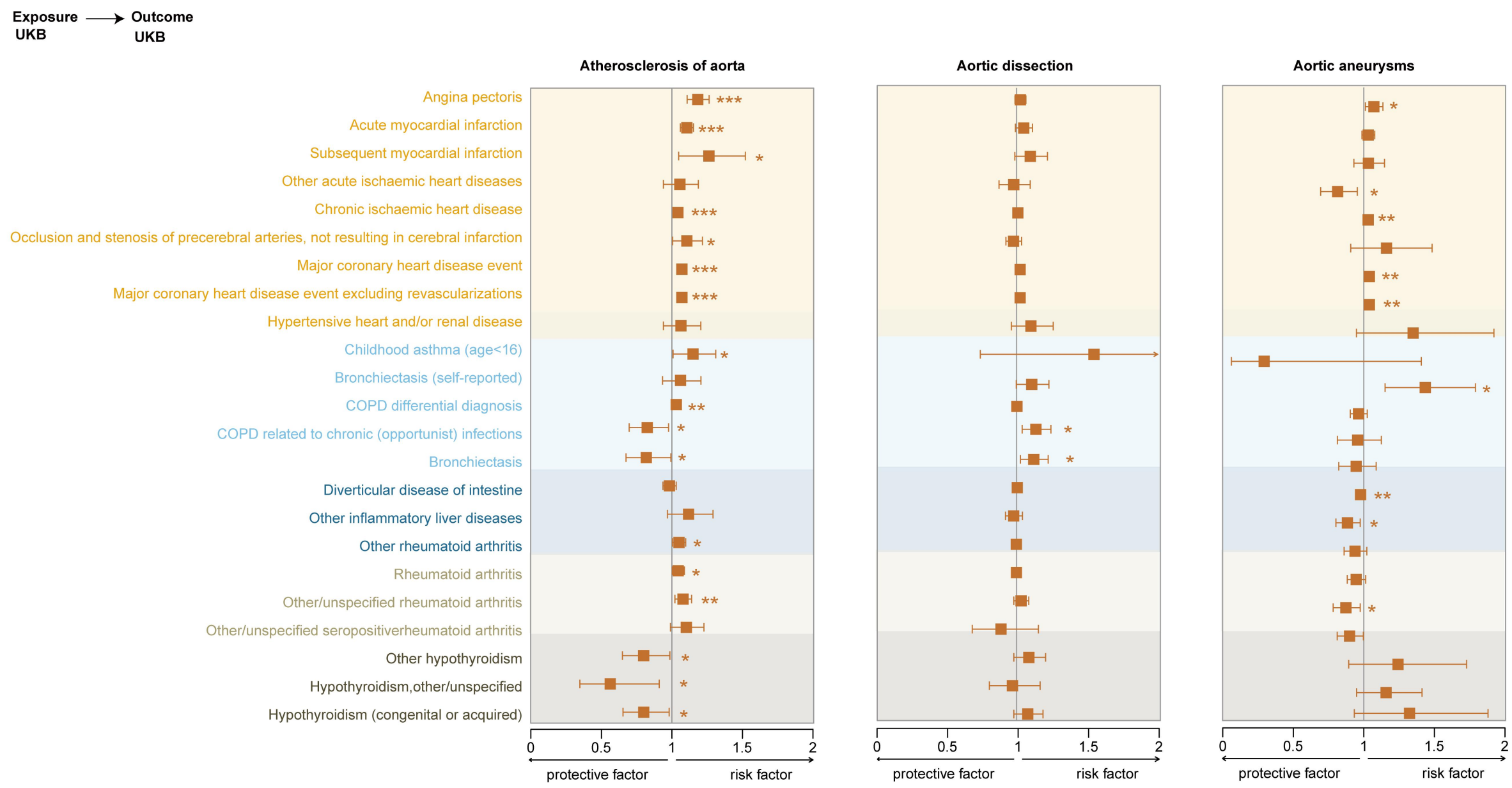

Supplementary Figure 3. Mendelian randomization analyses of genetically proxied chronic conditions and aortic disease outcomes in UK Biobank.

Forest plots show ORs and 95% CIs from MR analyses using UK Biobank summary statistics for both exposures and outcomes. Outcomes include aortic atherosclerosis, aortic dissection and aortic aneurysm. Points indicate OR estimates, and horizontal whiskers indicate 95% CIs. The vertical dashed line indicates the null value (OR = 1). *P* values were two-sided and were adjusted for multiple comparisons using the FDR method, where applicable. Exact *P* values and FDR-adjusted *P* values are provided in the corresponding Supplementary Data 7. Symbols indicate FDR-adjusted significance levels for visual reference: ***$P < 0.001$; **$P < 0.01$; *$P < 0.05$. Background shading indicates broad categories of exposure traits. No bar charts, representative blots, micrographs or gel images are shown. Abbreviations: OR, odds ratio; CI, confidence interval; COPD, chronic obstructive pulmonary disease.

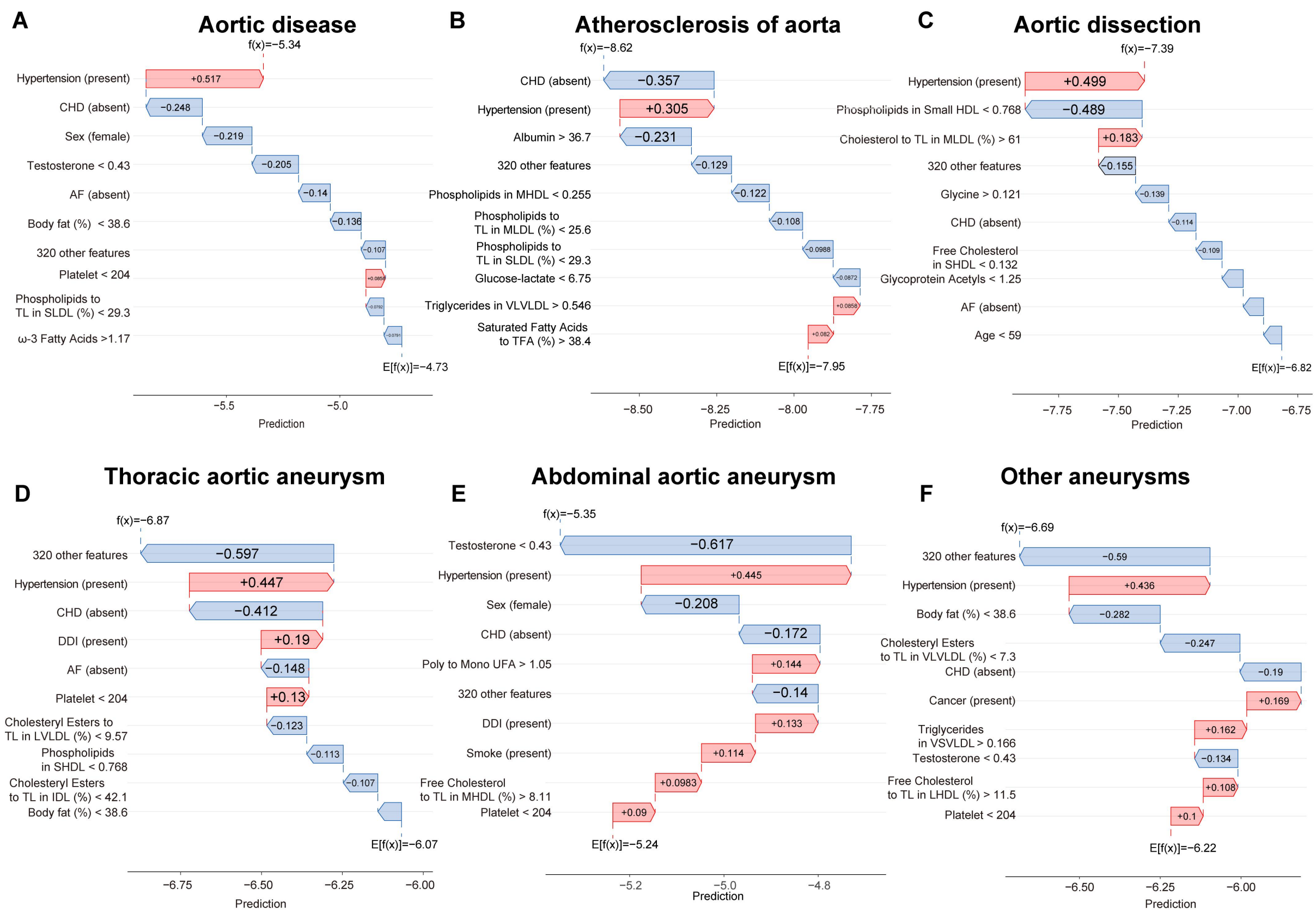

Supplementary Figure 4. SHAP force plots for representative individual predictions from XGBoost models in the UK Biobank.

SHAP force plots are shown for representative participants from XGBoost models predicting overall aortic disease (A), atherosclerosis of the aorta (B), aortic dissection (C), thoracic aortic aneurysm (D), abdominal aortic aneurysm (E) and other aneurysms (F). The models were developed in the UK Biobank analytical sample (n = 455,945 participants). The plots illustrate individual-level feature contributions for selected representative cases and are intended to show model interpretability rather than population-level statistical inference. Red features increase the model prediction, and blue features decrease the model prediction. The x axis represents the model prediction on the SHAP scale; f(x) indicates the individual prediction, and E[f(x)] indicates the expected model prediction. Abbreviations: SHAP, Shapley additive explanations; AF, atrial fibrillation; CHD, coronary heart disease; COPD, chronic obstructive pulmonary disease; CRP, C-reactive protein; HF, heart failure; PVD, peripheral vascular disease.

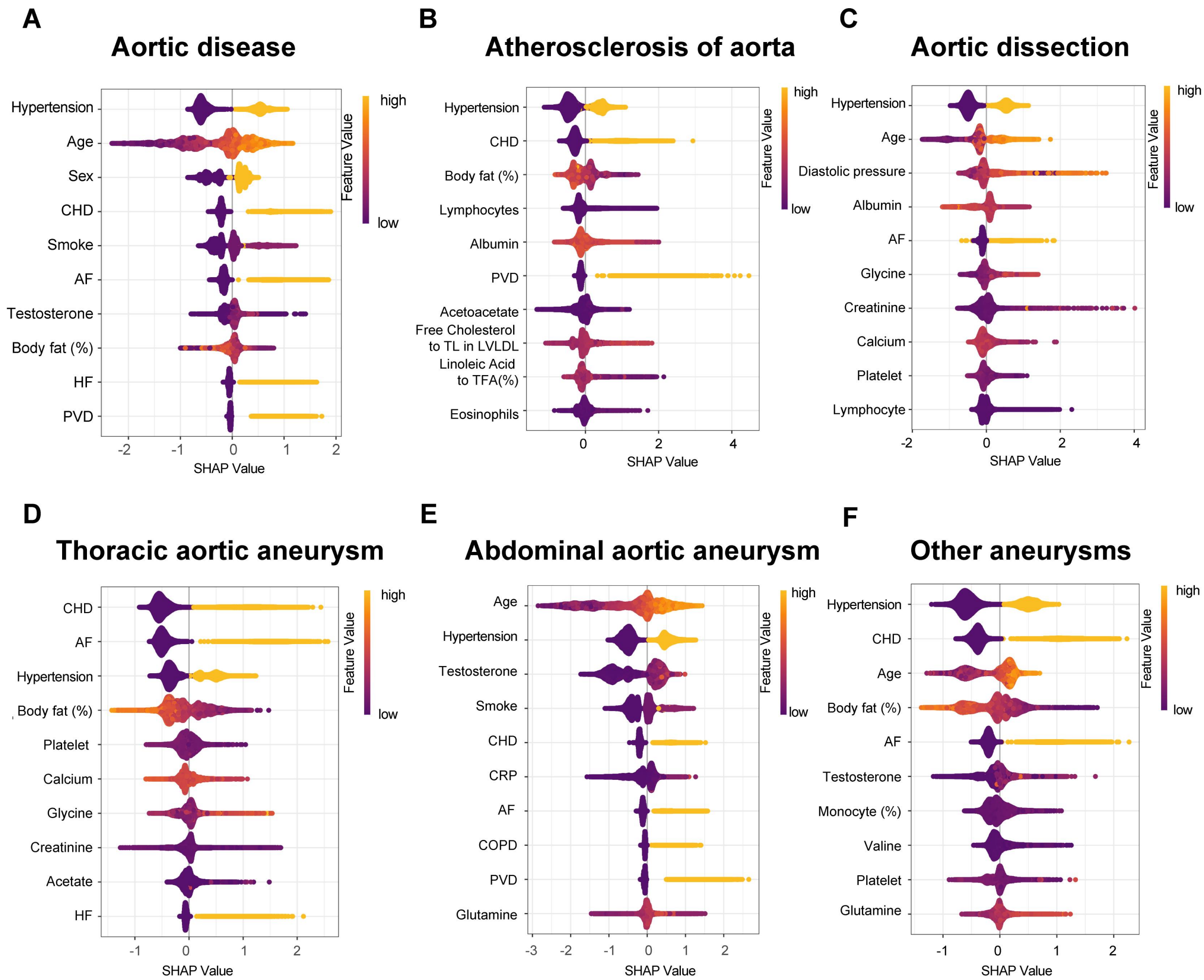

Supplementary Figure 5. Beeswarm plots of SHAP values for the top 10 features in the XGBoost models.

SHAP-based feature attribution plots are shown for XGBoost models developed in the UK Biobank analytical sample (n = 455,945 participants). The plots illustrate the contribution of model predictors to individual or model-level predictions for aortic disease outcomes. Colours, axes and symbols are defined in the figure panels and indicate the direction and magnitude of SHAP contributions. These plots are intended to describe model interpretability rather than formal hypothesis testing. Abbreviations: SHAP, Shapley additive explanations; AF, atrial fibrillation; CHD, coronary heart disease; COPD, chronic obstructive pulmonary disease; CRP, C-reactive protein; HF, heart failure; PVD, peripheral vascular disease.
