# Supplementary material for: Chronic conditions and aortic disease risk: a prospective cohort study with predictive and etiological analyses
Source: Nat Commun. 2026 Aug 11;17:9662. doi: 10.1038/s41467-026-76551-y (PMC13558640; doi:10.1038/s41467-026-76551-y)
Supplement: Supplementary file 2 [file 41467_2026_76551_MOESM2_ESM.pdf]

Description of Additional Supplementary Files

Supplementary Data 1. Associations of chronic conditions with overall and subtype-specific incident aortic diseases: Cox proportional hazards models in the UK Biobank.

Supplementary Data 2. Associations of chronic conditions with overall and subtype-specific incident aortic diseases: Cox proportional hazards models in the China Kadoorie Biobank.

Supplementary Data 3. Association of the number of chronic conditions with overall aortic disease incidence.

Supplementary Data 4. Estimated population attributable fractions for overall and subtype-specific aortic disease in UK Biobank.

Supplementary Data 5. Mendelian randomization analyses of genetically proxied chronic conditions and aortic disease outcomes in FinnGen.

Supplementary Data 6. Cross-dataset Mendelian randomization analyses of genetically proxied chronic conditions in FinnGen and aortic disease outcomes in UK Biobank.

Supplementary Data 7. Mendelian randomization analyses of genetically proxied chronic conditions and aortic disease outcomes in UK Biobank.

Supplementary Data 8. Sex-specific associations of chronic conditions with incident aortic disease in UK Biobank.

Supplementary Data 9. Sex-specific associations of chronic conditions with incident aortic atherosclerosis in UK Biobank.

Supplementary Data 10. Sex-specific associations of chronic conditions with incident aortic dissection in UK Biobank.

Supplementary Data 11. Sex-specific associations of chronic conditions with incident thoracic aortic aneurysm in UK Biobank.

Supplementary Data 12. Sex-specific associations of chronic conditions with incident abdominal aortic aneurysm in UK Biobank.

Supplementary Data 13. Sex-specific associations of chronic conditions with incident other aneurysms in UK Biobank.

Supplementary Data 14. Adjusted cause-specific hazard ratios for the association between chronic conditions and incident aortic disease in UK Biobank: competing-risk analysis.

Supplementary Data 15. Sensitivity analysis of the association between chronic conditions and incident aortic disease in UK Biobank using genetically inferred sex.

Supplementary Data 16. Sensitivity analysis of the association between chronic conditions and incident aortic dissection and aortic aneurysm in UK Biobank and China Kadoorie Biobank.

Supplementary Data 17. Sensitivity analysis of the associations between chronic conditions and incident aortic disease after excluding events occurring within the first 2 years of follow-up in UK Biobank and China Kadoorie Biobank.

Supplementary Data 18. Disease definitions, ICD-10 codes, and cohort-specific sample sizes for the 42 chronic conditions.

Supplementary Data 19. Definition of surgery for aortic disease in the UK Biobank.

Supplementary Data 20. Features incorporated into the XGBoost predictive model (n=329).
