# Supplementary material for: Chronic conditions and aortic disease risk: a prospective cohort study with predictive and etiological analyses
Source: Nat Commun. 2026 Aug 11;17:9662. doi: 10.1038/s41467-026-76551-y (PMC13558640; doi:10.1038/s41467-026-76551-y)
Supplement: Supplementary file 3 [file 41467_2026_76551_MOESM4_ESM.pdf]

nature portfolio

Corresponding author(s): Ding-Sheng Jiang, Xin Yi, Wei Liu and Dianjianyi Sun

Last updated by author(s): Jul 28, 2026

Reporting Summary

Nature Portfolio wishes to improve the reproducibility of the work that we publish. This form provides structure for consistency and transparency in reporting. For further information on Nature Portfolio policies, see our Editorial Policies and the Editorial Policy Checklist.

Statistics

For all statistical analyses, confirm that the following items are present in the figure legend, table legend, main text, or Methods section.

n/a	Confirmed	
☐	☒	The exact sample size (*n*) for each experimental group/condition, given as a discrete number and unit of measurement
☐	☒	A statement on whether measurements were taken from distinct samples or whether the same sample was measured repeatedly
☐	☒	The statistical test(s) used AND whether they are one- or two-sided<br>*Only common tests should be described solely by name; describe more complex techniques in the Methods section.*
☐	☒	A description of all covariates tested
☐	☒	A description of any assumptions or corrections, such as tests of normality and adjustment for multiple comparisons
☐	☒	A full description of the statistical parameters including central tendency (e.g. means) or other basic estimates (e.g. regression coefficient) AND variation (e.g. standard deviation) or associated estimates of uncertainty (e.g. confidence intervals)
☐	☒	For null hypothesis testing, the test statistic (e.g. *F*, *t*, *r*) with confidence intervals, effect sizes, degrees of freedom and *P* value noted<br>*Give P values as exact values whenever suitable.*
☒	☐	For Bayesian analysis, information on the choice of priors and Markov chain Monte Carlo settings
☒	☐	For hierarchical and complex designs, identification of the appropriate level for tests and full reporting of outcomes
☐	☒	Estimates of effect sizes (e.g. Cohen's *d*, Pearson's *r*), indicating how they were calculated

Our web collection on statistics for biologists contains articles on many of the points above.

Software and code

Policy information about availability of computer code

Data collection	No custom software or algorithms were developed by the authors for data collection. UK Biobank and China Kadoorie Biobank data were collected by the respective cohort teams using standardized cohort procedures, as described in the Methods.
Data analysis	All statistical analyses were performed in R version 4.5.1. Cox proportional hazards models were fitted using survival version 3.6.4. Mendelian randomization analyses were conducted using TwoSampleMR version 0.7.0 and ieugwasr version 1.1.0. Competing-risk analyses were conducted using riskRegression version 2025.9.17. Machine-learning analyses were conducted using xgboost version 1.7.11.1. Model interpretability was assessed using SHAP values computed with the built-in Tree SHAP implementation in xgboost and visualized using shapviz version 0.10.3. Data visualization was performed using ggplot2 version 3.5.1. The analysis code is publicly available at https://github.com/zhugecaoming/ukb-ckb-aortic-disease-code and has been archived as a published, version-specific Zenodo software record. DOI: https://doi.org/10.5281/zenodo.21620343.

For manuscripts utilizing custom algorithms or software that are central to the research but not yet described in published literature, software must be made available to editors and reviewers. We strongly encourage code deposition in a community repository (e.g. GitHub). See the Nature Portfolio guidelines for submitting code & software for further information.

Data

Policy information about availability of data

All manuscripts must include a data availability statement. This statement should provide the following information, where applicable:

- Accession codes, unique identifiers, or web links for publicly available datasets
- A description of any restrictions on data availability
- For clinical datasets or third party data, please ensure that the statement adheres to our policy

The summary-level data generated in this study are provided in Supplementary Table 1 and Supplementary Data files 1–20 accompanying this article and constitute the minimum dataset necessary to interpret, verify, and extend the findings. These data do not contain identifiable participant-level information.
The individual-level UK Biobank data used in this study are available under restricted access because they contain participant-level health, genetic, biomarker, and linked electronic health-record information governed by participant consent, data protection requirements, and UK Biobank data access policies. These data cannot be publicly deposited or redistributed by the authors. This study used UK Biobank data under Application ID 106528. Access can be obtained by bona fide researchers for approved health-related research through the UK Biobank Access Management System, subject to UK Biobank review and completion of relevant data access agreements. The response timeframe and duration of data availability after approval are determined by UK Biobank.
The individual-level China Kadoorie Biobank data used in this study are available under restricted access because they contain participant-level health information governed by participant consent, institutional approvals, data protection requirements, and CKB data access policies. These data cannot be publicly deposited or redistributed by the authors. Access can be obtained by qualified researchers for scientifically and ethically approved research through the China Kadoorie Biobank data access procedure [https://www.ckbiobank.org/data-access], subject to review and project-specific authorization by the relevant CKB data access committee and participating institutions. The expected response timeframe is up to 16 weeks in total, including formal application, review of proposal, approval, and signing of a Data Access Agreement. Data are available after approval for the period specified in the approved data access agreement or project authorization.
The GWAS summary statistics used in this study are available in the FinnGen database [https://r12.finngen.fi] and the UK Biobank summary statistics resource [https://pan.ukbb.broadinstitute.org].

Research involving human participants, their data, or biological material

Policy information about studies with human participants or human data. See also policy information about sex, gender (identity/presentation), and sexual orientation and race, ethnicity and racism.

Reporting on sex and gender	Sex was considered in the study design and analyses. In UK Biobank, sex was categorized as female or male, and in CKB as female or male based on cohort-recorded participant information. Sex was included as a covariate in the main adjusted models, and sex-stratified analyses were performed in UK Biobank to assess effect modification. In addition, sensitivity analyses were conducted using genetically inferred sex in place of self-reported sex in UK Biobank. The findings are reported as applying to both sexes, with sex-specific analyses reported where relevant. Gender-based information was not collected separately from sex in the datasets used.
Reporting on race, ethnicity, or other socially relevant groupings	In UK Biobank, ethnicity was included as a covariate and classified as White, Black or Black British, Asian or Asian British, Mixed heritage, or Other ethnic groups, based on cohort data. In CKB, participants were recruited from 10 geographically diverse regions of China, and region was included as a geographic covariate. Race/ethnicity was not a primary exposure of interest in this study, and no subgroup analyses were performed on this basis. These variables were included to account for potential confounding where applicable.
Population characteristics	This study included participants from two large prospective population-based cohorts. UK Biobank recruited adults aged 37–73 years between 2006 and 2010 from 22 assessment centres across England, Scotland and Wales. China Kadoorie Biobank recruited adults aged 30–79 years between 2004 and 2008 from 10 geographically diverse areas of China (five urban and five rural). The analytic sample comprised 455,945 participants in UK Biobank and 512,721 participants in CKB. Relevant participant characteristics considered in the analyses included age, sex, body mass index, smoking status, drinking status, educational level, household income, physical activity, and geographic region/assessment centre; ethnicity was additionally adjusted for in UK Biobank.
Recruitment	Participants were recruited through the established procedures of the UK Biobank and the China Kadoorie Biobank. UK Biobank recruited participants from 22 assessment centres across England, Scotland and Wales between 2006 and 2010. CKB recruited participants from 10 geographically diverse regions of China between 2004 and 2008. As with all volunteer-based cohort studies, some degree of self-selection may be present, and this may affect the representativeness of the study populations. The potential impact of cohort differences, including ancestry, age structure, disease ascertainment and healthcare context, is discussed in the manuscript.
Ethics oversight	UK Biobank: This research was conducted using the UK Biobank resource under Application ID 106528. UK Biobank has ethical approval from the NHS North West Multicentre Research Ethics Committee (REC reference: 16/NW/0274). Use of UK Biobank data complied with the provider's access requirements and the approved research application.<br>China Kadoorie Biobank: The CKB study was approved by the Oxford Tropical Research Ethics Committee (approval reference: 025-04), the Ethical Review Committee of the Chinese Center for Disease Control and Prevention, the Chinese Academy of Medical Sciences, and the Institutional Review Board of Peking University. Use of CKB data in the present study complied with the provider's access requirements, institutional approvals and data-use conditions.

Note that full information on the approval of the study protocol must also be provided in the manuscript.

Field-specific reporting

Please select the one below that is the best fit for your research. If you are not sure, read the appropriate sections before making your selection.

☒ Life sciences ☐ Behavioural & social sciences ☐ Ecological, evolutionary & environmental sciences

For a reference copy of the document with all sections, see nature.com/documents/nr-reporting-summary-flat.pdf

Life sciences study design

All studies must disclose on these points even when the disclosure is negative.

Sample size

No formal prospective sample-size calculation was performed because this was an observational analysis of existing biobank cohorts. Sample sizes were determined by the number of eligible participants available in UK Biobank and China Kadoorie Biobank after applying the predefined inclusion and exclusion criteria. The final analytical samples comprised 455,945 UKB participants and 512,721 CKB participants, with 4,354 and 5,329 incident aortic disease events, respectively. These large cohort sizes and event numbers were considered sufficient for the primary association analyses. Analyses with limited case numbers were interpreted cautiously, and conditions with insufficient exposed participants were excluded where appropriate.

Data exclusions

For each of the 42 chronic conditions, we excluded individuals with missing covariate data, those with prevalent aortic disease at baseline, those lost to follow-up, and those whose aortic disease onset preceded the chronic condition. For machine-learning analyses, participants with complete data for the selected variables were included.

Replication

The main observational associations were evaluated in two independent prospective cohorts: UK Biobank and China Kadoorie Biobank. Broadly consistent association patterns were observed across cohorts, supporting reproducibility of the main epidemiological findings. Additional robustness checks included Mendelian randomization analyses using external GWAS summary statistics, competing-risk analyses, analyses using genetically inferred sex, clinically specific endpoint analyses, and 2-year lag sensitivity analyses. Machine-learning models were internally evaluated using held-out test sets; external validation of the prediction models has not yet been performed. No experimental replication was performed because this was an observational cohort study using existing biobank data.

Randomization

This was an observational cohort study and no participants were randomized to experimental groups. In the machine-learning analyses, participants with complete data were randomly divided into a training set (70%) and a test set (30%) for model development and internal evaluation. Potential confounding in the observational analyses was addressed through multivariable adjustment for prespecified covariates.

Blinding

Blinding was not relevant to this observational cohort study because no experimental group allocation or investigator-administered intervention was involved. Outcome and exposure definitions were based on prespecified cohort and health-record data sources. For machine-learning analyses, model performance was evaluated in a held-out test set.

Behavioural & social sciences study design

All studies must disclose on these points even when the disclosure is negative.

Study description

Briefly describe the study type including whether data are quantitative, qualitative, or mixed-methods (e.g. qualitative cross-sectional, quantitative experimental, mixed-methods case study).

Research sample

State the research sample (e.g. Harvard university undergraduates, villagers in rural India) and provide relevant demographic information (e.g. age, sex) and indicate whether the sample is representative. Provide a rationale for the study sample chosen. For studies involving existing datasets, please describe the dataset and source.

Sampling strategy

Describe the sampling procedure (e.g. random, snowball, stratified, convenience). Describe the statistical methods that were used to predetermine sample size OR if no sample-size calculation was performed, describe how sample sizes were chosen and provide a rationale for why these sample sizes are sufficient. For qualitative data, please indicate whether data saturation was considered, and what criteria were used to decide that no further sampling was needed.

Data collection

Provide details about the data collection procedure, including the instruments or devices used to record the data (e.g. pen and paper, computer, eye tracker, video or audio equipment) whether anyone was present besides the participant(s) and the researcher, and whether the researcher was blind to experimental condition and/or the study hypothesis during data collection.

Timing

Indicate the start and stop dates of data collection. If there is a gap between collection periods, state the dates for each sample cohort.

Data exclusions

If no data were excluded from the analyses, state so OR if data were excluded, provide the exact number of exclusions and the rationale behind them, indicating whether exclusion criteria were pre-established.

Non-participation

State how many participants dropped out/declined participation and the reason(s) given OR provide response rate OR state that no participants dropped out/declined participation.

Randomization	*If participants were not allocated into experimental groups, state so OR describe how participants were allocated to groups, and if allocation was not random, describe how covariates were controlled.*

Ecological, evolutionary & environmental sciences study design

All studies must disclose on these points even when the disclosure is negative.

Study description	*Briefly describe the study. For quantitative data include treatment factors and interactions, design structure (e.g. factorial, nested, hierarchical), nature and number of experimental units and replicates.*
Research sample	*Describe the research sample (e.g. a group of tagged Passer domesticus, all Stenocereus thurberi within Organ Pipe Cactus National Monument), and provide a rationale for the sample choice. When relevant, describe the organism taxa, source, sex, age range and any manipulations. State what population the sample is meant to represent when applicable. For studies involving existing datasets, describe the data and its source.*
Sampling strategy	*Note the sampling procedure. Describe the statistical methods that were used to predetermine sample size OR if no sample-size calculation was performed, describe how sample sizes were chosen and provide a rationale for why these sample sizes are sufficient.*
Data collection	*Describe the data collection procedure, including who recorded the data and how.*
Timing and spatial scale	*Indicate the start and stop dates of data collection, noting the frequency and periodicity of sampling and providing a rationale for these choices. If there is a gap between collection periods, state the dates for each sample cohort. Specify the spatial scale from which the data are taken*
Data exclusions	*If no data were excluded from the analyses, state so OR if data were excluded, describe the exclusions and the rationale behind them, indicating whether exclusion criteria were pre-established.*
Reproducibility	*Describe the measures taken to verify the reproducibility of experimental findings. For each experiment, note whether any attempts to repeat the experiment failed OR state that all attempts to repeat the experiment were successful.*
Randomization	*Describe how samples/organisms/participants were allocated into groups. If allocation was not random, describe how covariates were controlled. If this is not relevant to your study, explain why.*
Blinding	*Describe the extent of blinding used during data acquisition and analysis. If blinding was not possible, describe why OR explain why blinding was not relevant to your study.*

Did the study involve field work? ☐ Yes ☐ No

Field work, collection and transport

Field conditions	*Describe the study conditions for field work, providing relevant parameters (e.g. temperature, rainfall).*
Location	*State the location of the sampling or experiment, providing relevant parameters (e.g. latitude and longitude, elevation, water depth).*
Access & import/export	*Describe the efforts you have made to access habitats and to collect and import/export your samples in a responsible manner and in compliance with local, national and international laws, noting any permits that were obtained (give the name of the issuing authority, the date of issue, and any identifying information).*
Disturbance	*Describe any disturbance caused by the study and how it was minimized.*

Reporting for specific materials, systems and methods

We require information from authors about some types of materials, experimental systems and methods used in many studies. Here, indicate whether each material, system or method listed is relevant to your study. If you are not sure if a list item applies to your research, read the appropriate section before selecting a response.

Materials & experimental systems

n/a	Involved in the study
☒	☐ Antibodies
☒	☐ Eukaryotic cell lines
☒	☐ Palaeontology and archaeology
☒	☐ Animals and other organisms
☒	☐ Clinical data
☒	☐ Dual use research of concern
☒	☐ Plants

Methods

n/a	Involved in the study
☒	☐ ChIP-seq
☒	☐ Flow cytometry
☒	☐ MRI-based neuroimaging

Antibodies

Antibodies used

Describe all antibodies used in the study; as applicable, provide supplier name, catalog number, clone name, and lot number.

Validation

Describe the validation of each primary antibody for the species and application, noting any validation statements on the manufacturer's website, relevant citations, antibody profiles in online databases, or data provided in the manuscript.

Eukaryotic cell lines

Policy information about cell lines and Sex and Gender in Research

Cell line source(s)

State the source of each cell line used and the sex of all primary cell lines and cells derived from human participants or vertebrate models.

Authentication

Describe the authentication procedures for each cell line used OR declare that none of the cell lines used were authenticated.

Mycoplasma contamination

Confirm that all cell lines tested negative for mycoplasma contamination OR describe the results of the testing for mycoplasma contamination OR declare that the cell lines were not tested for mycoplasma contamination.

Commonly misidentified lines (See ICLAC register)

Name any commonly misidentified cell lines used in the study and provide a rationale for their use.

Palaeontology and Archaeology

Specimen provenance

Provide provenance information for specimens and describe permits that were obtained for the work (including the name of the issuing authority, the date of issue, and any identifying information). Permits should encompass collection and, where applicable, export.

Specimen deposition

Indicate where the specimens have been deposited to permit free access by other researchers.

Dating methods

If new dates are provided, describe how they were obtained (e.g. collection, storage, sample pretreatment and measurement), where they were obtained (i.e. lab name), the calibration program and the protocol for quality assurance OR state that no new dates are provided.

☐ Tick this box to confirm that the raw and calibrated dates are available in the paper or in Supplementary Information.

Ethics oversight

Identify the organization(s) that approved or provided guidance on the study protocol, OR state that no ethical approval or guidance was required and explain why not.

Note that full information on the approval of the study protocol must also be provided in the manuscript.

Animals and other research organisms

Policy information about studies involving animals; ARRIVE guidelines recommended for reporting animal research, and Sex and Gender in Research

Laboratory animals

For laboratory animals, report species, strain and age OR state that the study did not involve laboratory animals.

Wild animals

Provide details on animals observed in or captured in the field; report species and age where possible. Describe how animals were caught and transported and what happened to captive animals after the study (if killed, explain why and describe method; if released, say where and when) OR state that the study did not involve wild animals.

Reporting on sex

Indicate if findings apply to only one sex; describe whether sex was considered in study design, methods used for assigning sex. Provide data disaggregated for sex where this information has been collected in the source data as appropriate; provide overall

	numbers in this Reporting Summary. Please state if this information has not been collected. Report sex-based analyses where performed, justify reasons for lack of sex-based analysis.
Field-collected samples	*For laboratory work with field-collected samples, describe all relevant parameters such as housing, maintenance, temperature, photoperiod and end-of-experiment protocol OR state that the study did not involve samples collected from the field.*
Ethics oversight	*Identify the organization(s) that approved or provided guidance on the study protocol, OR state that no ethical approval or guidance was required and explain why not.*

Note that full information on the approval of the study protocol must also be provided in the manuscript.

Clinical data

Policy information about clinical studies

All manuscripts should comply with the ICMJE guidelines for publication of clinical research and a completed CONSORT checklist must be included with all submissions.

Clinical trial registration	Not applicable. This study is an observational cohort study based on existing population-based biobank resources (UK Biobank and China Kadoorie Biobank) and is not a clinical trial.
Study protocol	The full study protocol for the present analysis is not separately publicly available. The study design, participant selection, exposure and outcome definitions, covariates, and statistical analysis methods are described in the manuscript. UK Biobank and China Kadoorie Biobank operate under their own established cohort protocols and governance frameworks.
Data collection	Data were collected within two established prospective population-based cohorts. UK Biobank recruited adults aged 37–73 years between 2006 and 2010 from 22 assessment centres across England, Scotland and Wales. China Kadoorie Biobank recruited adults aged 30–79 years between 2004 and 2008 from 10 geographically diverse areas of China (five urban and five rural). Baseline data collection included questionnaire-based assessment, physical measurements and blood sampling, with follow-up through linkage to electronic health records, including hospital records, death registries and related health data systems.
Outcomes	The primary outcome was incident aortic disease, ascertained using linked health record data in each cohort. In UK Biobank, case identification was based on national death registries, hospital inpatient diagnoses and aortic-related surgical procedure records. In CKB, outcomes were identified through linkage to death registries, disease registries and health insurance-based hospital records, with events centrally processed and coded using ICD-10. Aortic disease was defined using ICD-10 codes and included atherosclerosis of aorta (I70.0), aortic dissection (I71.0), thoracic aortic aneurysm with or without rupture (I71.1 and I71.2), abdominal aortic aneurysm with or without rupture (I71.3 and I71.4), other aortic aneurysms (I71.5, I71.6, I71.8 and I71.9), and inflammatory aortic arch syndrome (M31.4). Subtype-specific analyses were also performed.

Dual use research of concern

Policy information about dual use research of concern

Hazards

Could the accidental, deliberate or reckless misuse of agents or technologies generated in the work, or the application of information presented in the manuscript, pose a threat to:

No	Yes	
☐	☐	Public health
☐	☐	National security
☐	☐	Crops and/or livestock
☐	☐	Ecosystems
☐	☐	Any other significant area

Experiments of concern

Does the work involve any of these experiments of concern:

No	Yes	
☐	☐	Demonstrate how to render a vaccine ineffective
☐	☐	Confer resistance to therapeutically useful antibiotics or antiviral agents
☐	☐	Enhance the virulence of a pathogen or render a nonpathogen virulent
☐	☐	Increase transmissibility of a pathogen
☐	☐	Alter the host range of a pathogen
☐	☐	Enable evasion of diagnostic/detection modalities
☐	☐	Enable the weaponization of a biological agent or toxin
☐	☐	Any other potentially harmful combination of experiments and agents

Plants

Seed stocks	Not applicable; no plants or plant materials were used in this study.
Novel plant genotypes	Not applicable; no plants or plant genotypes were used in this study.
Authentication	Not applicable; no plants or plant materials were used in this study.

ChIP-seq

Data deposition

☐ Confirm that both raw and final processed data have been deposited in a public database such as GEO.

☐ Confirm that you have deposited or provided access to graph files (e.g. BED files) for the called peaks.

Data access links<br>*May remain private before publication.*	*For "Initial submission" or "Revised version" documents, provide reviewer access links. For your "Final submission" document, provide a link to the deposited data.*
Files in database submission	*Provide a list of all files available in the database submission.*
Genome browser session<br>(e.g. UCSC)	*Provide a link to an anonymized genome browser session for "Initial submission" and "Revised version" documents only, to enable peer review. Write "no longer applicable" for "Final submission" documents.*

Methodology

Replicates	*Describe the experimental replicates, specifying number, type and replicate agreement.*
Sequencing depth	*Describe the sequencing depth for each experiment, providing the total number of reads, uniquely mapped reads, length of reads and whether they were paired- or single-end.*
Antibodies	*Describe the antibodies used for the ChIP-seq experiments; as applicable, provide supplier name, catalog number, clone name, and lot number.*
Peak calling parameters	*Specify the command line program and parameters used for read mapping and peak calling, including the ChIP, control and index files used.*
Data quality	*Describe the methods used to ensure data quality in full detail, including how many peaks are at FDR 5% and above 5-fold enrichment.*
Software	*Describe the software used to collect and analyze the ChIP-seq data. For custom code that has been deposited into a community repository, provide accession details.*

Flow Cytometry

Plots

Confirm that:

- [ ] The axis labels state the marker and fluorochrome used (e.g. CD4-FITC).
- [ ] The axis scales are clearly visible. Include numbers along axes only for bottom left plot of group (a 'group' is an analysis of identical markers).
- [ ] All plots are contour plots with outliers or pseudocolor plots.
- [ ] A numerical value for number of cells or percentage (with statistics) is provided.

Methodology

Sample preparation	*Describe the sample preparation, detailing the biological source of the cells and any tissue processing steps used.*
Instrument	*Identify the instrument used for data collection, specifying make and model number.*
Software	*Describe the software used to collect and analyze the flow cytometry data. For custom code that has been deposited into a community repository, provide accession details.*
Cell population abundance	*Describe the abundance of the relevant cell populations within post-sort fractions, providing details on the purity of the samples and how it was determined.*
Gating strategy	*Describe the gating strategy used for all relevant experiments, specifying the preliminary FSC/SSC gates of the starting cell population, indicating where boundaries between "positive" and "negative" staining cell populations are defined.*

- [ ] Tick this box to confirm that a figure exemplifying the gating strategy is provided in the Supplementary Information.

Magnetic resonance imaging

Experimental design

Design type	*Indicate task or resting state; event-related or block design.*
Design specifications	*Specify the number of blocks, trials or experimental units per session and/or subject, and specify the length of each trial or block (if trials are blocked) and interval between trials.*
Behavioral performance measures	*State number and/or type of variables recorded (e.g. correct button press, response time) and what statistics were used to establish that the subjects were performing the task as expected (e.g. mean, range, and/or standard deviation across subjects).*

Acquisition

Imaging type(s)	*Specify: functional, structural, diffusion, perfusion.*
Field strength	*Specify in Tesla*
Sequence & imaging parameters	*Specify the pulse sequence type (gradient echo, spin echo, etc.), imaging type (EPI, spiral, etc.), field of view, matrix size, slice thickness, orientation and TE/TR/flip angle.*
Area of acquisition	*State whether a whole brain scan was used OR define the area of acquisition, describing how the region was determined.*

Diffusion MRI ☐ Used ☐ Not used

Preprocessing

Preprocessing software	*Provide detail on software version and revision number and on specific parameters (model/functions, brain extraction, segmentation, smoothing kernel size, etc.).*
Normalization	*If data were normalized/standardized, describe the approach(es): specify linear or non-linear and define image types used for transformation OR indicate that data were not normalized and explain rationale for lack of normalization.*
Normalization template	*Describe the template used for normalization/transformation, specifying subject space or group standardized space (e.g. original Talairach, MNI305, ICBM152) OR indicate that the data were not normalized.*
Noise and artifact removal	*Describe your procedure(s) for artifact and structured noise removal, specifying motion parameters, tissue signals and physiological signals (heart rate, respiration).*

Volume censoring | *Define your software and/or method and criteria for volume censoring, and state the extent of such censoring.*

Statistical modeling & inference

Model type and settings | *Specify type (mass univariate, multivariate, RSA, predictive, etc.) and describe essential details of the model at the first and second levels (e.g. fixed, random or mixed effects; drift or auto-correlation).*

Effect(s) tested | *Define precise effect in terms of the task or stimulus conditions instead of psychological concepts and indicate whether ANOVA or factorial designs were used.*

Specify type of analysis: ☐ Whole brain ☐ ROI-based ☐ Both

Statistic type for inference | *Specify voxel-wise or cluster-wise and report all relevant parameters for cluster-wise methods.*

(See Eklund et al. 2016)

Correction | *Describe the type of correction and how it is obtained for multiple comparisons (e.g. FWE, FDR, permutation or Monte Carlo).*

Models & analysis

n/a	Involved in the study
☐	☐ Functional and/or effective connectivity
☐	☐ Graph analysis
☐	☐ Multivariate modeling or predictive analysis

Functional and/or effective connectivity | *Report the measures of dependence used and the model details (e.g. Pearson correlation, partial correlation, mutual information).*

Graph analysis | *Report the dependent variable and connectivity measure, specifying weighted graph or binarized graph, subject- or group-level, and the global and/or node summaries used (e.g. clustering coefficient, efficiency, etc.).*

Multivariate modeling and predictive analysis | *Specify independent variables, features extraction and dimension reduction, model, training and evaluation metrics.*
