# Supplementary material for: Chronic conditions and aortic disease risk: a prospective cohort study with predictive and etiological analyses
Source: Nat Commun. 2026 Aug 11;17:9662. doi: 10.1038/s41467-026-76551-y (PMC13558640; doi:10.1038/s41467-026-76551-y)
Supplement: Supplementary file 4 [file 41467_2026_76551_MOESM5_ESM.pdf]

nature portfolio

Peer Review File

Chronic Conditions and Aortic Disease Risk: A Prospective Cohort Study with Predictive and Etiological Analyses

Corresponding Author: Dr Ding-Sheng Jiang

This file contains all reviewer reports in order by version, followed by all author rebuttals in order by version.

Version 0:

Reviewer comments:

Reviewer #1

(Remarks to the Author)
Well done study, however, authors need to make sure their source code is public and available with processed dataset to validate and reproduce their results. Furthermore, they must attach a supplementary material (user guide) to explain non-expert users to how to utilize their source and apply to their processed dataset.

Reviewer #2

(Remarks to the Author)
-Some sentences in the background are not logically correct. For example, in the following sentence of "Beyond their individual effects, chronic diseases may interact to promote vascular degeneration. For example, diabetes predisposes to multiple complications, including cardiomyopathy and diabetic retinopathy7. Similarly, left-sided heart disease (e.g., left ventricular failure) and lung disease (e.g., chronic obstructive pulmonary disease [COPD]) may synergistically contribute to pulmonary hypertension and vascular remodeling8.", diabetic vasculopathy is the result of the disease and the relationship are well appreciated. The following sentence does not fit to explain these relationships. "Beyond their individual effects, chronic diseases may interact to promote vascular degeneration."

-"Additionally, we developed interpretable machine learning models using Shapley Additive exPlanations (SHAP) to evaluate predictive accuracy and identify dominant clinical and biochemical risk factors." Please explain more about this analysis. Needs reference as well.

-Software used for MR analysis needs to be written. Also, the MR analysis codes need to be included in supplemental files.

-In the Cox regression, did the author conduct multivariable analyses? Did the author consider collinearity between variables?

-Number of each aortic outcomes needs be written in the manuscript.

-Similar chronic conditions were related to opposite effect to aortic outcomes, and this leads impression of unstable analysis, probably linked to low number of outcomes. This is limitation in this study and findings of this study needs to be carefully considered. In this sense, the impact to clinical practice from this study is quite limited.

-Some chronic condition is not chronic. For example, acute alcohol intoxication or pneumonia is not chronic.

-Availability of data needs to be disclose to the readership. "Summary-level genome-wide association data" needs to be included in the manuscript. Also, appropriate reference is missing.

-Figure 2. The same color needs to be used for a disease across 6 figures within the Figure 2 to avoid confusions. For example, COPD needs to be yellow in all 6 figures.

-Figure 5. In SHAP plots, meanings of variables are not clear currently. For example, what is the meaning of AF =1? Also, Body Fat 37.3%, what does it mean? I believe there should be > or < for these values.

Reviewer #3

(Remarks to the Author)
The authors should be commended on their comprehensive analysis of associations between 42 chronic conditions and incident aortic disease in 452,786 UK Biobank participants over 14.6 years of follow-up. While the scale and scope of this investigation, as well as the triangulation of evidence approach, are all major strengths of this study, there remain substantial methodological limitations that preclude the use of strong causal language in this manuscript. Specifically:
- In the Mendelian randomisation analysis, the authors used a genome-wide significance threshold of $P < 5\times10^{-5}$ for instrument selection, which is substantially more lenient than the conventional $P < 5\times10^{-8}$. This markedly increases the risk of weak instrument bias and false-positive findings. Preferably, the conventional threshold should be used, otherwise justification for this lenient threshold is required
- Along a similar line, F-statistics for instrument strength are not reported. Conventional approaches would accept only values >10 for use as instruments
- The source of exposure GWAS data is unclear. If FinnGen was used for both exposures and outcomes, this introduces sample overlap bias that would inflate MR estimates toward confounded observational associations.
- The use of causal language is overstated. The title claims chronic conditions "drive" aortic disease, and the abstract states MR analyses "supported causal effects." However, observational associations predominate, and the MR results show modest effect sizes with methodological limitations. The manuscript would benefit from more measured causal language throughout.
- PAF calculations assume a causal relationship between exposure and outcome. Given that causality is not definitively established for most conditions examined, these estimates should be interpreted with appropriate caveats. The statement that hypertension "accounted for" 50% of aortic disease cases implies causation that may not be warranted for all exposures.
- Only internal validation was performed. Given the well-known risk of overfitting with XGBoost and the high training set AUROCs (0.93–0.98), external validation in an independent cohort is essential before claims of "clinical utility" can be substantiated. Moreover the discrepancy between training and test AUROCs for some subtypes (e.g. aortic dissection: 0.963 vs 0.741) raises concerns about generalisability.
- There are also potential biases introduced via the exposure ascertainment approach. Chronic conditions were ascertained via self-report at baseline. This introduces potential misclassification bias, particularly for conditions with variable diagnostic accuracy (e.g. IBS, chronic fatigue syndrome). The authors acknowledge this limitation but should quantify potential impact through sensitivity analyses using hospital diagnostic codes where available.

Version 1:

Reviewer comments:

Reviewer #1

(Remarks to the Author)
Authors tried to address my comment.

Reviewer #2

(Remarks to the Author)
The author addressed to the comments to the reviewers in this revision.
I still think that there is significant concern in this study.
As this cohort study's outcome is incidence of aortic disease, the absence of the aortic disease needs to be clarified.
Especially, "Atherosclerosis of aorta" (I70.0) is vague medical condition. It is quite common that patients have plaque without any symptoms. Setting this as a part of outcome is source of bias. About 30% and 80% of overall aortic disease in UKB and CKB is I70.0.
The absence of outcome at baseline need be proved.
Using I70.0 is source of bias.

Reviewer #3

(Remarks to the Author)
Thank you for addressing the previous comments.

Version 2:

Reviewer comments:

Reviewer #2

(Remarks to the Author)
The authors made revisions based on reviewers comments. Thank you.

Open Access This Peer Review File is licensed under a Creative Commons Attribution 4.0 International License, which permits use, sharing, adaptation, distribution and reproduction in any medium or format, as long as you give appropriate credit to the original author(s) and the source, provide a link to the Creative Commons license, and indicate if changes were made.
In cases where reviewers are anonymous, credit should be given to 'Anonymous Referee' and the source.
The images or other third party material in this Peer Review File are included in the article's Creative Commons license, unless indicated otherwise in a credit line to the material. If material is not included in the article's Creative Commons license and your intended use is not permitted by statutory regulation or exceeds the permitted use, you will need to obtain permission directly from the copyright holder.
To view a copy of this license, visit https://creativecommons.org/licenses/by/4.0/

Response to Reviewers

Manuscript ID: [NCOMMS-25-95985A]

Reviewer #1:

Well done study, however, authors need to make sure their source code is public and available with processed dataset to validate and reproduce their results. Furthermore, they must attach a supplementary material (user guide) to explain non-expert users to how to utilize their source and apply to their processed dataset.

Response (R1): We sincerely thank the reviewer for the recognition and high appreciation of our study, as well as for the constructive comments provided. In response, we have taken the following steps.

First, we have made the analysis code publicly available through **GitHub** (https://github.com/zhugecaoming/ukb-ckb-aortic-disease-code) and have revised the **Code Availability** statement accordingly. The public repository includes the main analysis scripts used in this study, together with a README and script-level instructions for code navigation.

Second, with regard to the processed dataset, we respectfully note that the individual-level data used in this study were obtained under controlled-access agreements from the UK Biobank (UKB) and the China Kadoorie Biobank (CKB). Under the corresponding data access and ethical governance frameworks, we are not permitted to publicly redistribute either the raw individual-level data or derived processed individual-level datasets. For this reason, we are unable to make the processed dataset publicly available. We have clarified in the **Data Availability** statement that qualified researchers may apply for access to the underlying data directly through the official access procedures of UKB and CKB, subject to institutional approval and project-specific authorization.

Third, in direct response to the Reviewer's request, we have added a **Supplementary User Guide** to help non-expert users understand how to navigate the public code package, prepare local inputs, and run the main workflow. This guide describes the repository structure, software requirements, local folder setup, expected input files, variable dictionaries, step-by-step analysis workflow, and the distinction between

materials that can be shared publicly and those that must remain under controlled access.

These revisions have now been incorporated into the manuscript in the **Code Availability** and **Data Availability** sections, and the **Supplementary User Guide** has been added to the Supplementary Materials.

Reviewer #2:

Comment 1 (C1): Some sentences in the background are not logically correct. For example, in the following sentence of "Beyond their individual effects, chronic diseases may interact to promote vascular degeneration. For example, diabetes predisposes to multiple complications, including cardiomyopathy and diabetic retinopathy7. Similarly, left-sided heart disease (e.g., left ventricular failure) and lung disease (e.g., chronic obstructive pulmonary disease [COPD]) may synergistically contribute to pulmonary hypertension and vascular remodeling8.", diabetic vasculopathy is the result of the disease and the relationship are well appreciated. The following sentence does not fit to explain these relationships. "Beyond their individual effects, chronic diseases may interact to promote vascular degeneration."

Response (R1): We thank the Reviewer for this careful and helpful comment. In response, we have revised the Introduction to remove this problematic wording and the specific examples cited by the Reviewer. Instead, we now use a more general and more precise formulation, stating that multimorbidity may capture cumulative vascular, metabolic, inflammatory, and haemodynamic stress that is not fully reflected by individual risk factors considered in isolation. This revised wording better reflects the rationale for studying multimorbidity in relation to aortic disease without implying unsupported mechanistic relationships. These changes have been incorporated in the Introduction.

Page 5, line 92

Multimorbidity, commonly defined as the coexistence of two or more chronic conditions, is now highly prevalent in adult and especially older populations, with substantial variation across settings but consistently increasing with age, affecting over one-third of adults globally[1]. In this context, multimorbidity may capture cumulative vascular, metabolic, inflammatory and haemodynamic stress that is not fully reflected by individual risk factors considered in isolation.

Comment 2 (C2): "Additionally, we developed interpretable machine learning models using Shapley Additive exPlanations (SHAP) to evaluate predictive accuracy and identify dominant clinical and biochemical risk factors." Please explain more about this analysis. Needs reference as well.

Response (R2): We thank the Reviewer for this helpful suggestion. In response, we have revised the manuscript to provide a clearer and more detailed description of this analysis. Specifically, we now clarify that XGBoost-based prediction models were developed in UK Biobank (UKB) for overall and subtype-specific aortic disease using 329 baseline demographic, clinical, chronic disease, and laboratory variables, that participants were randomly divided into training (70%) and test (30%) sets, and that predictors were ranked within the training set according to XGBoost gain, with the top 10 predictors retained for the final parsimonious models. We further specify that model performance was evaluated using the area under the receiver operating characteristic curve (ROC-AUC).

We have also expanded the explanation of Shapley Additive exPlanations (SHAP). The revised text now states that SHAP was used to quantify the contribution of each predictor to model output at both the global level (overall feature importance across participants) and the individual level (feature contributions for representative predictions). This clarification has also been reflected in the Results and figure legends, where we now more explicitly describe the SHAP-based feature attribution analyses. These revisions are now reflected in the **Methods**, and **Results**. In addition, we have added the appropriate methodological **references** for both XGBoost and SHAP, including Chen and Guestrin (2016), Lundberg and Lee (2017), and Lundberg et al. (2020)[1-3].

<u>**Page 27, line 635**</u>

Machine learning model establishment and feature selection

Machine learning models were developed in UKB to predict overall and subtype-specific aortic disease using the XGBoost algorithm. Candidate predictors included 329 baseline demographic, clinical, chronic disease, and laboratory variables routinely available in UKB (**Supplementary Table S15**). Participants with complete data for the selected variables were randomly divided into a training set (70%) and a test set (30%). Within the training set, features were ranked according to XGBoost gain, and the top 10 predictors for each outcome were retained to construct parsimonious final models. Final XGBoost models were then trained in the training set and evaluated in the held-out test set. Model discrimination was assessed using the area under the receiver operating characteristic curve (ROC-AUC). To improve interpretability, we applied Shapley Additive exPlanations (SHAP) to quantify the

contribution of each predictor to model output at both the global level (overall feature importance across participants) and the individual level (feature contributions for representative predictions). Global importance was summarized by aggregating the absolute SHAP values across individuals, and local explanations were visualized using SHAP force plots for representative participants[33, 34, 35].

<u>Page 12, line 261</u>

Machine learning-based prediction of aortic disease

We next developed XGBoost-based prediction models in UKB for overall and subtype-specific aortic disease using the top 10 predictors ranked by XGBoost gain. The model for overall aortic disease showed good discrimination, with an AUROC of 0.880 (95% CI 0.874–0.886) in the training set and 0.860 (0.851–0.870) in the internal test set. Subtype-specific models also performed well, with training-set AUROCs of 0.984 for aortic atherosclerosis, 0.963 for aortic dissection, 0.948 for thoracic aortic aneurysm, 0.932 for abdominal aortic aneurysm, and 0.956 for other aneurysms, whereas the corresponding test-set AUROCs ranged from 0.741 to 0.885, indicating variable but generally favorable internal performance across phenotypes (**Figure 4**).

Shapley additive explanations (SHAP) analyses identified hypertension as the most influential predictor across models, together with age, sex, coronary heart disease, atrial fibrillation, and smoking, while several laboratory markers contributed differentially across subtype-specific models. In the subtype-specific models, albumin and diastolic pressure were prominent for aortic dissection, whereas platelet count, creatinine, C-reactive protein, and several lipid-related measures contributed to discrimination for selected aneurysm phenotypes. Individual-level SHAP force plots further illustrated substantial heterogeneity in feature contributions across representative cases, supporting model interpretability at both the global and individual levels (**Supplementary Figures S4–S5**).

References

[1] Chen T, Guestrin C. XGBoost: A Scalable Tree Boosting System. In: *Proceedings of the 22nd ACM SIGKDD International Conference on Knowledge Discovery and Data Mining*). Association for Computing Machinery (2016).

[2] Lundberg SM, Lee S-I. A unified approach to interpreting model predictions. In:

Proceedings of the 31st International Conference on Neural Information Processing Systems. Curran Associates Inc. (2017).

[3] Lundberg SM, *et al.* From Local Explanations to Global Understanding with Explainable AI for Trees. *Nat Mach Intell* **2**, 56-67 (2020).

Comment 3 (C3): Software used for MR analysis needs to be written. Also, the MR analysis codes need to be included in supplemental files.

Response (R3): Thank you for this insightful suggestion. Following the reviewer's comment, we have now clarified the software used for the Mendelian randomization (MR) analyses in the Methods section and made the corresponding MR analysis code publicly available to improve reproducibility.

Specifically, all MR analyses were conducted using **R software (version 4.3.1)**. The main MR analyses were implemented using the **TwoSampleMR** package (version 0.5.7), which was used for data harmonization and for the primary MR methods described in the manuscript, including **inverse variance weighted (IVW)**, **MR-Egger**, **weighted median**, and **weighted mode** analyses. Where applicable, GWAS summary data management was performed using the **ieugwasr** package.

In addition, the MR analysis scripts have now been uploaded to our public **GitHub** repository: https://github.com/zhugecaoming/ukb-ckb-aortic-disease-code.

We have also updated the **Code Availability** statement accordingly. To facilitate code navigation, the repository includes documentation for the MR-related scripts, and a corresponding **Supplementary User Guide** has been provided to help readers understand the workflow, required inputs, and outputs. These revisions have now been incorporated into the Methods, Code Availability, and Supplementary Materials sections of the revised manuscript.

Page 27, line 629

Mendelian randomization analyses were conducted in R software (version 4.3.1) using the *TwoSampleMR* package (version 0.5.7) for data harmonization and implementation of the main MR methods, with *ieugwasr* used where applicable for GWAS summary data management. Associations were considered statistically significant at P <0.05, and consistency across datasets and methods was taken into account in interpretation.

Comment 4 (C4): In the Cox regression, did the author conduct multivariable analyses? Did the author consider collinearity between variables?

Response (R4): Thank you for this important question. Yes, we conducted multivariable Cox proportional hazards regression analyses in both the UK Biobank (UKB) and the China Kadoorie Biobank (CKB), with CKB newly incorporated as an independent validation cohort.

In UKB, hazard ratios (HRs) and 95% confidence intervals (CIs) were estimated with adjustment for age, sex, assessment centre, ethnicity, smoking status, body mass index (BMI), drinking status, educational level, household income, and physical activity. In CKB, the corresponding multivariable models were adjusted for age, sex, region, smoking status, BMI, drinking status, educational level, household income, and physical activity.

We also assessed potential collinearity among the covariates included in the multivariable models. **Variance inflation factors (VIFs)** were calculated, and all covariates showed **VIF < 2**, indicating no evidence of problematic multicollinearity. Therefore, no covariates were excluded on the basis of collinearity, and the multivariable estimates are unlikely to have been materially influenced by collinearity. We have clarified this in the revised Methods section.

<u>**Page 25, line 583**</u>

Multicollinearity among the adjusted covariates was evaluated using variance inflation factors, and all covariates showed variance inflation factors < 2, indicating no evidence of problematic collinearity.

Comment 5 (C5): Number of each aortic outcomes needs be written in the manuscript.

Response (R5): Thank you for this helpful suggestion. In response, we have revised the manuscript to explicitly report the numbers of overall and subtype-specific aortic disease events in both UKB and CKB. Specifically, we now state in the Results section that, during follow-up, 4,354 participants in UKB and 5,329 participants in CKB developed overall aortic disease, and we additionally provide the corresponding numbers for atherosclerosis of aorta, aortic dissection, thoracic aortic aneurysm, abdominal aortic aneurysm, and other aneurysms. These event counts are also

summarized in **Supplementary Table S1**. We further clarify that, because participants could contribute to more than one subtype category, subtype-specific event counts do not necessarily sum to the total number of overall aortic disease cases.

Table S1. Numbers of overall and subtype-specific aortic disease events in the UK Biobank and China Kadoorie Biobank

**Outcome**	**UKB, n**	**CKB, n**
Overall aortic disease	4,354	5,329
Atherosclerosis of aorta	1,423	4,075
Aortic dissection	792	501
Thoracic aortic aneurysm	1,133	387
Abdominal aortic aneurysm	2,392	436
Other aneurysms	1,184	249

Note: Because participants could contribute to more than one subtype category, subtype-specific event counts do not necessarily sum to the total number of overall aortic disease cases.

Comment 6 (C6): Similar chronic conditions were related to opposite effect to aortic outcomes, and this leads impression of unstable analysis, probably linked to low number of outcomes. This is limitation in this study and findings of this study needs to be carefully considered. In this sense, the impact to clinical practice from this study is quite limited.

Response (R6): Thank you for this thoughtful and important comment. We agree that some chronic conditions showed heterogeneous associations across different aortic disease outcomes, and that this could create an impression of instability, particularly in subtype-specific analyses with relatively small numbers of events.

In response, we have revised the manuscript to address this issue more explicitly and cautiously. First, we now emphasize that aortic disease comprises clinically heterogeneous phenotypes, including aortic atherosclerosis, dissection, thoracic aneurysm, abdominal aneurysm, and other aneurysms, and that differences in the pattern of association across these outcomes may therefore reflect genuine biological and clinical heterogeneity rather than a single uniform disease process. Second, we

agree that for some subtype-specific outcomes, especially those with smaller event counts, the corresponding estimates should be interpreted with caution because reduced precision may contribute to apparent variability in effect size and, in some instances, direction.

Importantly, we also note in the revised manuscript that some heterogeneity in subtype-specific associations was observed across both UKB and CKB, suggesting that these patterns may not be attributable solely to statistical instability, but may also reflect differences in the underlying pathophysiology and aetiology of distinct aortic disease subtypes. At the same time, we acknowledge that limited event numbers for some subtype-specific outcomes reduce precision and warrant cautious interpretation.

We have therefore strengthened the Discussion and Limitations sections to avoid overstating the immediate clinical implications of subtype-specific findings. We now emphasize that the most robust message of the study lies in the overall pattern observed across two large cohorts, namely, that chronic disease burden, including both cardiovascular and selected non-cardiovascular conditions, is associated with aortic disease risk, whereas the subtype-specific findings should be interpreted as more exploratory and hypothesis-generating.

Accordingly, we have revised the manuscript to present the subtype-specific findings more cautiously and to better acknowledge the limitations related to outcome heterogeneity, event number, and the limited immediate clinical applicability of some subtype-level associations.

Page 16, line 374

Although some subtype-specific estimates should be interpreted cautiously because of limited event numbers, similar heterogeneity was observed across UKB and CKB, suggesting that the variation may not be due solely to statistical instability but may also reflect differences in the pathophysiology of distinct aortic disease subtypes.

Page 19, line 442

Fourth, some subtype-specific analyses involved relatively small case numbers, which may have reduced precision for selected estimates and contributed to heterogeneity in the corresponding associations. Although some of these subtype-specific patterns were observed across both cohorts, they should still be interpreted cautiously and require further confirmation.

Comment 7 (C7): Some chronic condition is not chronic. For example, acute

alcohol intoxication or pneumonia is not chronic.

Response (R7): Thank you for this important comment. We agree that some ICD-10 categories initially used in the manuscript were overly broad and could include acute or non-chronic conditions. To address this concern, we have revised the disease definitions and restricted them to chronic or recurrent forms wherever appropriate.

Specifically, we changed viral hepatitis from B15–B19 to B18 (chronic viral hepatitis); alcohol problems from F10 to F10.1, F10.2, F10.6, F10.7, F10.8, thereby excluding acute alcohol intoxication (F10.0); other psychoactive substance misuse from F19 to F19.1, F19.2, F19.8, F19.9; painful condition from R52 to R52.1 and R52.2 (chronic pain conditions); and we removed N13.2 from the definition of chronic kidney disease. These revisions have been incorporated into the **Supplementary TableS13** describing ICD-10 code definitions, and the manuscript has been updated accordingly.

Comment 8 (C8): Availability of data needs to be disclose to the readership. "Summary-level genome-wide association data" needs to be included in the manuscript. Also, appropriate reference is missing.

Response (R8): Thank you for this important comment. In response, we have revised the manuscript to more clearly disclose the availability and provenance of the data used in this study, including the summary-level genome-wide association study (GWAS) datasets used for the Mendelian randomization analyses.

Specifically, we have revised the Methods section to state explicitly that the MR analyses were based on summary-level GWAS data from FinnGen and UK Biobank-derived GWAS resources. We have also added the corresponding references for these datasets in the revised manuscript, including Kurki et al[1]. for FinnGen and Karczewski et al. for the Pan-UK Biobank resource[2].

In addition, we have expanded the Data Availability statement to clarify access conditions for both the individual-level cohort data (UK Biobank and China Kadoorie Biobank) and the summary-level GWAS data used for the MR analyses. The revised manuscript now states that the individual-level UKB and CKB data are available through controlled-access application procedures, whereas the GWAS summary statistics from FinnGen and UK Biobank/Pan-UK Biobank are available through their corresponding public resources.

These revisions are now reflected in the Methods, Data Availability, and References sections of the revised manuscript.

Page 25, line 599

Mendelian randomization analysis

To assess whether selected associations between chronic conditions and aortic disease were supported by genetic evidence, we performed MR analyses using summary-level genome-wide association study (GWAS) data from FinnGen and UK Biobank-derived GWAS resources[31, 32].

Data Availability

The GWAS summary statistics are available with unrestricted access. The GWAS summary statistics from FinnGen are available at https://r12.finngen.fi. The GWAS summary statistics from UK Biobank are available at https://pan.ukbb.broadinstitute.org.

Reference

[1] Kurki MI, et al. FinnGen provides genetic insights from a well-phenotyped isolated population. Nature 613, 508-518 (2023).

[2] Karczewski KJ, et al. Pan-UK Biobank genome-wide association analyses enhance discovery and resolution of ancestry-enriched effects. Nat Genet 57, 2408-2417 (2025).

Comment 9 (C9): Figure 2. The same color needs to be used for a disease across 6 figures within the Figure 2 to avoid confusions. For example, COPD needs to be yellow in all 6 figures.

Response (R9): Thank you for this helpful suggestion. In response, we have revised Figure 3 (previous Figure 2) to use a consistent color scheme for each chronic condition across all six sub-panels. Each disease is now represented by the same color in every panel to improve visual consistency and reduce ambiguity when comparing results across aortic disease outcomes. For example, COPD is now shown in yellow throughout all six panels of new **Figure 3**.

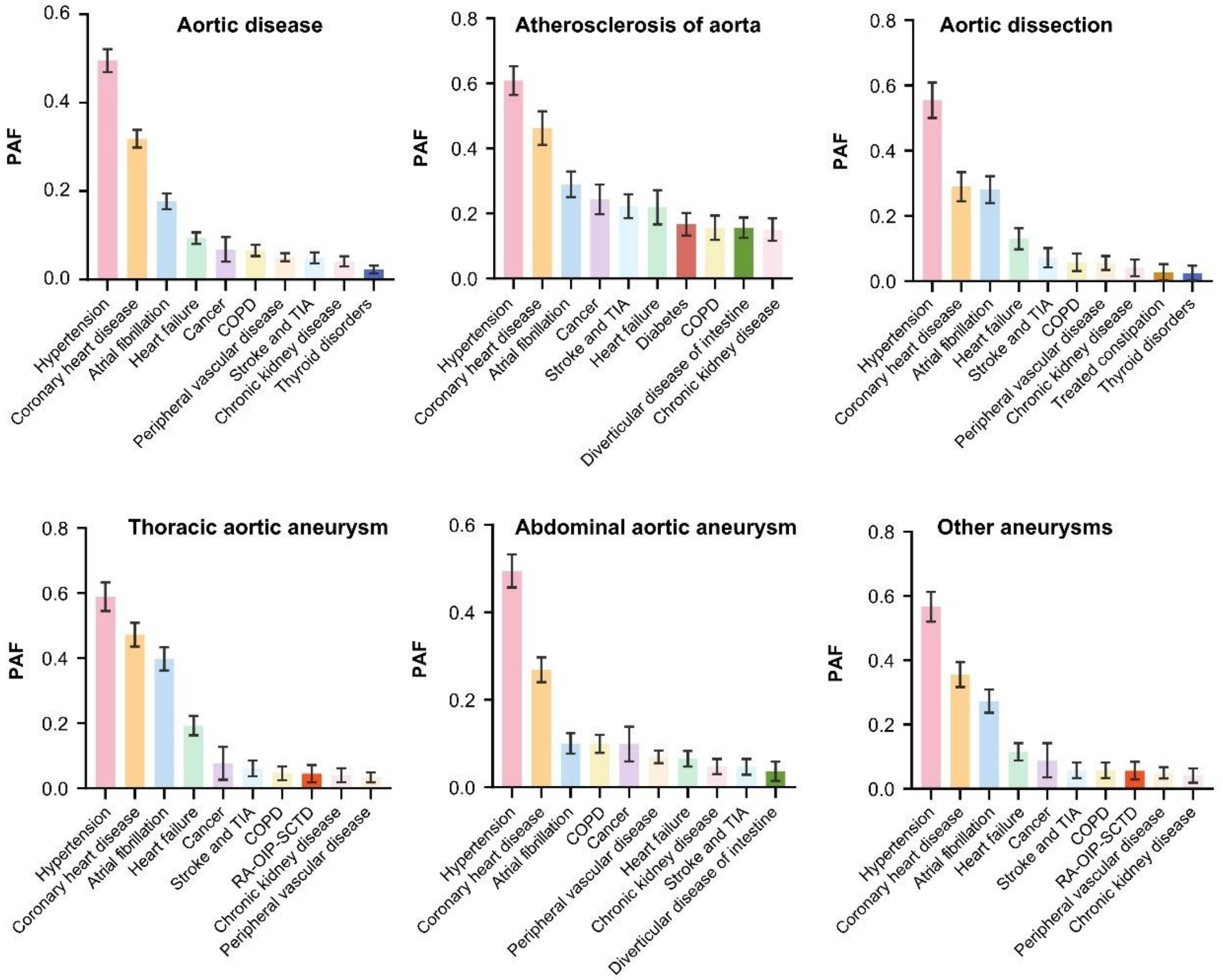

Figure 3. Population attributable fractions for overall and subtype-specific aortic disease in UK Biobank.

Comment 10 (C10): Figure 5. In SHAP plots, meanings of variables are not clear currently. For example, what is the meaning of AF =1? Also, Body Fat 37.3%, what does it mean? I believe there should be > or < for these values.

Response (R10): Thank you for this constructive comment regarding the clarity of the variable labels in the SHAP plots.

In response, we have revised **Figure 4** (previous Figure 5) to make the SHAP force plots more intuitive and interpretable. Specifically:

- Binary variables are now labeled using clinically meaningful wording rather than numeric coding. For example, “AF = 1” has been replaced by “AF (present)”, and corresponding negative states are shown as “AF (absent)”. Similar revisions were made for other binary variables such as hypertension, CHD, COPD, HF, and PVD.
- Continuous variables are now displayed with explicit directional symbols (“>” or

“<”) to indicate whether the feature value is above or below the representative threshold shown in the SHAP plot. For example, values are now presented as “Body fat (%) < 37.3”, “Albumin > 39.2”, and “Age < 51”, which more clearly indicate the direction of the feature contribution to the model prediction.

- We have also updated the Figure 4 legend to clarify that the SHAP force plots display representative feature values and their directional contributions to the model output.

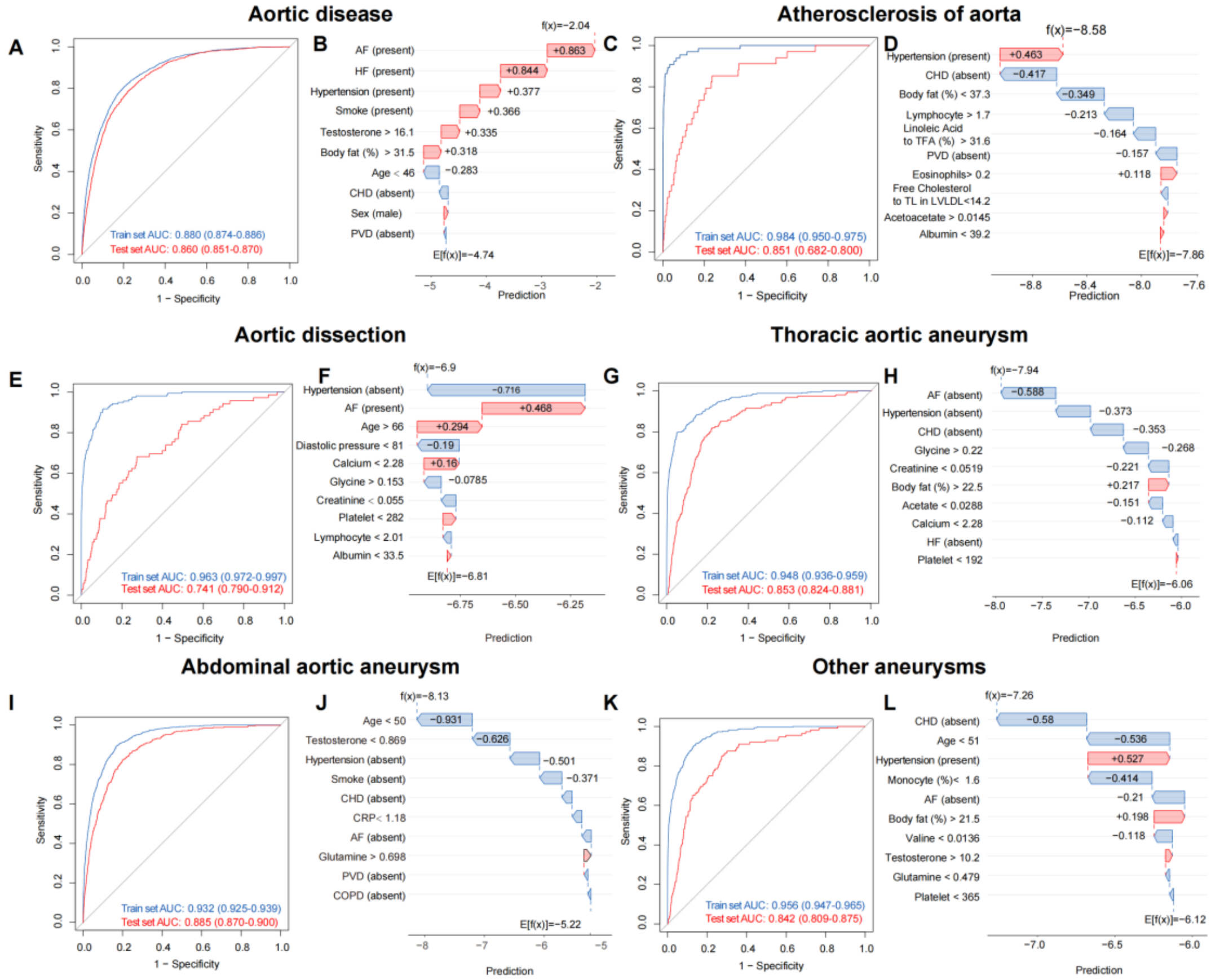

Figure 4. Predictive performance and feature attribution of XGBoost models for aortic disease and its subtypes in UK Biobank.

Reviewer #3:

The authors should be commended on their comprehensive analysis of associations between 42 chronic conditions and incident aortic disease in 452,786 UK Biobank participants over 14.6 years of follow-up. While the scale and scope of this investigation, as well as the triangulation of evidence approach, are all major strengths of this study, there remain substantial methodological limitations that preclude the use of strong causal language in this manuscript. Specifically:

Comment 1 (C1): In the Mendelian randomisation analysis, the authors used a genome-wide significance threshold of $P < 5\times10^{-5}$ for instrument selection, which is substantially more lenient than the conventional $P < 5\times10^{-8}$. This markedly increases the risk of weak instrument bias and false-positive findings. Preferably, the conventional threshold should be used, otherwise justification for this lenient threshold is required.

Response (R1): We sincerely thank the reviewer for the valuable comments regarding the selection of instrumental variables.

In response, we have revised both the analyses and the manuscript text to clarify our instrument-selection strategy and to interpret the findings more cautiously. Specifically, for UK Biobank (UKB) summary statistics, we used the conventional genome-wide significance threshold ($P < 5\times10^{-8}$) and retained variants with F-statistic > 10 as instrumental variables. For FinnGen, however, many clinically defined chronic disease traits yielded very few or no eligible variants under the conventional threshold, likely reflecting substantial phenotypic and genetic heterogeneity across these conditions. To balance instrument availability with instrument strength, we therefore applied a more pragmatic strategy in FinnGen: variants were initially selected at $P < 5\times10^{-6}$ with F-statistic ≥ 10, and when fewer than five eligible variants were available, the threshold was further relaxed to $P < 5\times10^{-5}$, while requiring F-statistic ≥ 15.

Importantly, we now make explicit in the revised manuscript that the UKB analyses using the conventional threshold serve as the more conservative reference, whereas the FinnGen-based analyses should be interpreted more cautiously, particularly for traits with limited cross-dataset consistency. We have also revised the Results and Discussion to avoid overstatement and to emphasize that these MR analyses provide supportive genetic evidence rather than definitive causal proof.

These revisions are now reflected in the Methods, Results, Discussion, and the relevant MR figures and supplementary materials.

Page 25, line 599

Mendelian randomization analysis

To assess whether selected associations between chronic conditions and aortic disease were supported by genetic evidence, we performed MR analyses using summary-level genome-wide association study (GWAS) data from FinnGen and UK Biobank-derived GWAS resources[31, 32]. Genetic instruments for each chronic condition were selected from the corresponding GWAS summary statistics. In UKB, we used the conventional genome-wide significance threshold ($P < 5\times10^{-8}$) and retained variants with F-statistic > 10 as instrumental variables. In FinnGen, because several disease traits yielded only a limited number of eligible variants under the conventional threshold, likely reflecting phenotypic and genetic heterogeneity across clinically defined disease categories, a pragmatic thresholding strategy was applied. The initial selection threshold was set at $P < 5\times10^{-6}$, with exclusion of weak instruments (F-statistic < 10). When fewer than five eligible variants were available under this threshold, the significance threshold was further relaxed to $P < 5\times10^{-5}$, while requiring F-statistic ≥ 15 to retain instrument strength. Independent variants were identified by linkage disequilibrium clumping ($r^2 < 0.001$, 10,000-kb window).

Page 11, line 241

Genetic analyses using Mendelian randomization

To provide genetic support for selected observational associations, we performed Mendelian randomization (MR) analyses using summary-level data from FinnGen and UKB. Across analyses, genetically predicted hypertension showed the most consistent associations with multiple aortic disease outcomes, including aortic atherosclerosis, aortic dissection, and thoracic and abdominal aortic aneurysm phenotypes (**Supplementary Figures S1-S3)**. Major coronary heart disease also demonstrated positive associations with several aortic outcomes, particularly aortic atherosclerosis and thoracic aortic aneurysm. Importantly, the genetic signals were not confined to cardiometabolic traits: COPD- and asthma-related phenotypes also showed evidence of association with selected aneurysm outcomes across several analyses, and additional signals were observed for selected non-cardiovascular traits in specific datasets. Additional analyses using different exposure–outcome data sources yielded broadly similar patterns, while also indicating heterogeneity across

datasets and disease definitions. Overall, these genetic analyses provide supportive evidence that the observed aortic disease risk profile extends beyond traditional cardiovascular conditions and includes selected non-cardiovascular traits, particularly respiratory phenotypes, although the strength and consistency of these associations varied across outcomes and datasets.

Page 17, line 394

In FinnGen, several clinically defined chronic conditions yielded few genome-wide significant variants under conventional thresholds, and the use of a more pragmatic instrument-selection strategy increased trait coverage but may also have introduced greater uncertainty into some estimates. Accordingly, these MR findings should be interpreted primarily as supportive rather than definitive, particularly for traits showing limited cross-dataset consistency.

Comment 2 (C2): Along a similar line, F-statistics for instrument strength are not reported. Conventional approaches would accept only values >10 for use as instruments.

Response (R2): We thank the reviewer for this important methodological comment regarding the assessment of instrumental variable strength. We completely agree with the reviewer that the F-statistic is a critical indicator for evaluating weak instrument bias, and the conventional threshold ($F > 10$) is essential for ensuring the reliability of Mendelian randomization analyses.

In our initial analysis, although we did not explicitly report the F-statistics in the manuscript, all instrumental variables retained after screening had F-statistics well above the conventional threshold of 10. However, we acknowledge that this information should have been presented transparently.

To further enhance the rigor and credibility of our findings, and in line with the more stringent screening strategy described in our response to Comment C1, we have now re-performed the instrumental variable selection process with an additional filter. Specifically, after selecting genetic variants based on the significance thresholds (as detailed in our previous response), we further excluded any weak instruments with an F-statistic <15. We then re-conducted the entire two-sample Mendelian randomization analysis using this more robust set of instruments.

Encouragingly, the results obtained from this stricter pipeline remained consistent

with our original findings and the updated results presented in response to Comment C1. The significant comorbid effects of hypertension, CHD, COPD, and asthma on aortic disease were still observed in both the FinnGen and UK Biobank databases. These consistent results across different thresholds further demonstrate the robustness of our conclusions. We have now added the F-statistic calculations and this additional sensitivity analysis to the revised manuscript.

Page 26, line 603

Genetic instruments for each chronic condition were selected from the corresponding GWAS summary statistics. In UKB, we used the conventional genome-wide significance threshold ($P < 5\times10^{-8}$) and retained variants with F-statistic > 10 as instrumental variables. In FinnGen, because several disease traits yielded only a limited number of eligible variants under the conventional threshold, likely reflecting phenotypic and genetic heterogeneity across clinically defined disease categories, a pragmatic thresholding strategy was applied. The initial selection threshold was set at $P < 5\times10^{-6}$, with exclusion of weak instruments (F-statistic < 10). When fewer than five eligible variants were available under this threshold, the significance threshold was further relaxed to $P < 5\times10^{-5}$, while requiring F-statistic ≥ 15 to retain instrument strength. Independent variants were identified by linkage disequilibrium clumping (r^2 < 0.001, 10,000-kb window).

Comment 3 (C3): The source of exposure GWAS data is unclear. If FinnGen was used for both exposures and outcomes, this introduces sample overlap bias that would inflate MR estimates toward confounded observational associations.

Response (R3): We thank the reviewer for raising this important methodological concern regarding potential sample overlap bias. We agree that, when exposure and outcome GWAS summary statistics are derived from the same underlying dataset, overlap between samples may bias Mendelian randomization (MR) estimates toward the corresponding observational associations.

In the revised manuscript, we now explicitly state that the MR analyses used summary-level GWAS data from **FinnGen** and **UK Biobank**-derived GWAS resources, and we clarify the corresponding exposure–outcome data pairings in the Methods, Data Availability, and References sections.

To address the Reviewer's concern, we further clarify that our MR analyses were

conducted in multiple stages. We first performed MR analyses within each available data source to assess whether selected observational associations were supported by genetically proxied effects. Because such within-dataset analyses may be more susceptible to overlap-related bias, we interpreted them cautiously. We then conducted additional cross-dataset MR analyses using exposure and outcome summary statistics derived from different sources, thereby substantially reducing concern about sample overlap bias.

Importantly, the major signals, particularly those involving hypertension and coronary heart disease, and to some extent COPD- and asthma-related phenotypes, remained broadly consistent in the cross-dataset analyses. We therefore interpret these findings as supportive evidence that the main MR signals were not solely driven by sample overlap, while still acknowledging the remaining limitations of the MR framework.

These clarifications are now reflected in the revised **Methods** (pages 25–26), **Results** (pages 11–12), **Data Availability** (pages 29–30), **Discussion** (pages 16–17) sections, and **Supplementary Figures S1-S3** of the manuscript.

Comment 4 (C4): The use of causal language is overstated. The title claims chronic conditions "drive" aortic disease, and the abstract states MR analyses "supported causal effects." However, observational associations predominate, and the MR results show modest effect sizes with methodological limitations. The manuscript would benefit from more measured causal language throughout.

Response (R4): We thank the Reviewer for this important comment. We have revised the manuscript throughout to adopt a more cautious and proportionate interpretation of the findings. Specifically:

- **Title**

  We revised the title to remove overly strong causal wording. In particular, the previous wording implying that chronic conditions "drive" aortic disease has been replaced with more neutral language focused on risk, prediction, and etiological analyses.

- **Abstract**

  We removed wording suggesting that the MR analyses demonstrated "causal effects" and replaced it with more measured phrasing, stating that the MR

analyses provided genetic support for selected associations, particularly for hypertension and coronary heart disease.

- **Results**

  We revised the MR results section to avoid causal overstatement. The revised text now emphasizes that the MR findings provide supportive evidence and that the observed signals extend beyond traditional cardiovascular traits, while also acknowledging variability across datasets and outcomes.

- **Discussion and Conclusion**

  We have revised these sections to ensure that the observational findings are interpreted as associations, not proof of causation, and that the MR analyses are described as providing supportive genetic evidence rather than definitive causal proof. We also explicitly acknowledge the methodological limitations of the MR analyses, including heterogeneity in phenotype definition, variable instrument strength, and the use of a more pragmatic instrument-selection strategy for some FinnGen traits.

These changes are reflected in the revised Title, Abstract, Results, Discussion, and Conclusion.

Title

Chronic Conditions and Aortic Disease Risk: A Prospective Cohort Study with Predictive and Etiological Analyses

Page 3, line 65

Mendelian randomization analyses provided genetic support for selected associations, particularly for hypertension and coronary heart disease. Machine learning models integrating chronic conditions with routinely collected demographic, clinical, and laboratory variables demonstrated good performance in predicting the risk of overall and subtype-specific aortic disease.

Page 11, line 241

Genetic analyses using Mendelian randomization

To provide genetic support for selected observational associations, we performed Mendelian randomization (MR) analyses using summary-level data from FinnGen and UKB. Across analyses, genetically predicted hypertension showed the most consistent associations with multiple aortic disease outcomes, including aortic atherosclerosis, aortic dissection, and thoracic and abdominal aortic aneurysm phenotypes (**Supplementary Figures S1-S3)**. Major coronary heart disease also

demonstrated positive associations with several aortic outcomes, particularly aortic atherosclerosis and thoracic aortic aneurysm. Importantly, the genetic signals were not confined to cardiometabolic traits: COPD- and asthma-related phenotypes also showed evidence of association with selected aneurysm outcomes across several analyses, and additional signals were observed for selected non-cardiovascular traits in specific datasets. Additional analyses using different exposure–outcome data sources yielded broadly similar patterns, while also indicating heterogeneity across datasets and disease definitions. Overall, these genetic analyses provide supportive evidence that the observed aortic disease risk profile extends beyond traditional cardiovascular conditions and includes selected non-cardiovascular traits, particularly respiratory phenotypes, although the strength and consistency of these associations varied across outcomes and datasets.

Page 17, line 394

In FinnGen, several clinically defined chronic conditions yielded few genome-wide significant variants under conventional thresholds, and the use of a more pragmatic instrument-selection strategy increased trait coverage but may also have introduced greater uncertainty into some estimates. Accordingly, these MR findings should be interpreted primarily as supportive rather than definitive, particularly for traits showing limited cross-dataset consistency.

Comment 5 (C5): PAF calculations assume a causal relationship between exposure and outcome. Given that causality is not definitively established for most conditions examined, these estimates should be interpreted with appropriate caveats. The statement that hypertension "accounted for" 50% of aortic disease cases implies causation that may not be warranted for all exposures.

Response (R5): We thank the Reviewer for this important comment. In response, we have revised the manuscript to use more cautious language when describing the PAF results. Specifically, we now refer to these estimates as the estimated population-attributable burden or estimated population-attributable fraction, rather than implying definitive causal attribution. For example, wording such as hypertension "accounted for" 50% of cases has been revised to indicate that hypertension had the largest estimated attributable fraction under the assumptions of

the PAF framework.

These changes are now reflected in the revised Results and relevant figure legends.

Page 3, line 62

In UKB, hypertension showed the largest estimated population-attributable fraction across aortic disease phenotypes, while other chronic conditions, including COPD and chronic kidney disease, also contributed to the estimated attributable burden.

Page 10, line 219

For overall aortic disease, hypertension had the largest estimated population-attributable fraction (49.5%, 95% CI, 46.9–52.1%).

Page 11, line 236

Overall, these findings indicate that in UKB, hypertension had the largest estimated attributable burden, with additional contributions from coronary heart disease, atrial fibrillation, and selected non-cardiovascular conditions depending on disease subtype.

Figure legends

Figure 3. Estimated population attributable fractions for overall and subtype-specific aortic disease in UK Biobank.

Comment 6 (C6): Only internal validation was performed. Given the well-known risk of overfitting with XGBoost and the high training set AUROCs (0.93–0.98), external validation in an independent cohort is essential before claims of "clinical utility" can be substantiated. Moreover the discrepancy between training and test AUROCs for some subtypes (e.g. aortic dissection: 0.963 vs 0.741) raises concerns about generalisability.

Response (R6): We thank the Reviewer for this important comment. We have revised the manuscript in several ways.

First, we now state more explicitly in the Results, Discussion, and Limitations that the machine learning models were developed and evaluated using internal validation only within UK Biobank (UKB), based on a training/test split, and that no independent external validation cohort was available for the prediction models. Although the epidemiological association analyses were externally evaluated in China Kadoorie Biobank (CKB), the prediction models themselves were not externally validated because the machine learning workflow relied on a set of detailed baseline demographic, clinical, and laboratory variables that were not fully harmonized across

cohorts.

Second, we have moderated the language regarding model performance and clinical relevance. We no longer present these models as having established clinical utility. Instead, we now describe them as showing good internal predictive performance for overall aortic disease and more variable performance across subtype-specific outcomes, while emphasizing that their clinical applicability remains to be determined through independent external validation.

Third, we now acknowledge more explicitly that the discrepancy between training and test AUROCs for some outcomes, for example, aortic dissection, may reflect a degree of overfitting and/or reduced stability related to the smaller number of subtype-specific events. We therefore interpret these subtype-specific prediction results more cautiously in the revised Discussion.

Finally, we emphasize that future work should validate these models in independent cohorts and assess their transportability across populations before any clinical implementation is considered.

These revisions are now reflected in the revised manuscript.

Page 13, line 282

Overall, machine learning models integrating chronic conditions with routinely collected variables showed good internal performance for predicting overall and subtype-specific aortic disease, with interpretable feature attribution. These findings suggest that routinely available clinical information may be useful for future risk stratification of aortic disease; however, independent external validation will be required before any clinical application can be considered.

Page 19, line 449

Finally, the prediction models were developed and internally validated only in UKB. The observed train–test performance gaps for some subtype-specific models suggest possible overfitting or limited transportability, and independent external validation is required before clinical application.

Comment 7 (C7): There are also potential biases introduced via the exposure ascertainment approach. Chronic conditions were ascertained via self-report at baseline. This introduces potential misclassification bias, particularly for conditions with variable diagnostic accuracy (e.g. IBS, chronic fatigue syndrome). The authors acknowledge this limitation but should quantify potential impact

through sensitivity analyses using hospital diagnostic codes where available.

Response (R7): We thank the Reviewer for this important comment regarding potential exposure misclassification. We would like to clarify that, in the UK Biobank (UKB) analyses, chronic conditions were not ascertained by participant self-report. Instead, they were defined using linked ICD-10 diagnosis records from hospital inpatient data (UK Biobank Field 41270). Therefore, the primary UKB exposure definitions were based on externally sourced diagnostic records rather than self-reported baseline illness data.

We agree, however, that the source and quality of chronic condition ascertainment are important considerations, and that misclassification remains possible, particularly for conditions with heterogeneous clinical definitions or incomplete capture in routine health records. In the revised manuscript, we have therefore made the source of chronic condition ascertainment in UKB more explicit in the Methods section. We have also revised the Discussion to acknowledge that chronic conditions were defined using cohort-specific health data sources in both UKB and CKB, and may still be subject to some degree of misclassification or under-ascertainment, particularly for subclinical or undiagnosed disease.

Because the primary UKB analyses were already based on hospital ICD-10 diagnostic records rather than self-reported exposures, the requested sensitivity analysis comparing self-reported versus hospital-based ascertainment was not applicable to the main UKB analyses. However, we have clarified this point in the revised manuscript to ensure that the data source and its limitations are accurately represented.

These revisions are now reflected in the Methods and Discussion sections of the revised manuscript.

<u>Page 21, line 500</u>

Chronic conditions were defined using cohort-specific health data sources and harmonized into a common analytical framework. In the UKB, chronic conditions were ascertained from linked ICD-10 diagnosis records (Field 41270), derived from hospital inpatient data. In the CKB, chronic conditions were ascertained using available baseline survey data and linked electronic health records, including disease registries and health insurance records, and were harmonized to the same disease categories as those used in UKB wherever possible.

<u>Page 19, line 440</u>

Third, ascertainment of chronic conditions relied on cohort-specific health data sources and may still be subject to misclassification or under-ascertainment, particularly for subclinical or undiagnosed disease.

Response to Reviewers

Manuscript ID: [NCOMMS-25-95985A]

Reviewer #1:

I am satisfied with the revision.

Response: We sincerely thank the reviewer for their positive evaluation and are pleased that the revisions have addressed their concerns.

Reviewer #2:

Comment 1 (C1): The author addressed to the comments to the reviewers in this revision. I still think that there is significant concern in this study. As this cohort study's outcome is incidence of aortic disease, the absence of the aortic disease needs to be clarified. Especially, "Atherosclerosis of aorta" (I70.0) is vague medical condition. It is quite common that patients have plaque without any symptoms. Setting this as a part of outcome is source of bias. About 30% and 80% of overall aortic disease in UKB and CKB is I70.0. The absence of outcome at baseline need be proved. Using I70.0 is source of bias.

Response: We thank the reviewer for this important and thoughtful comment. We agree that atherosclerosis of aorta coded as I70.0 is a relatively broad administrative diagnosis and may include asymptomatic, incidental, or pre-existing aortic atherosclerosis detected during clinical care. To address this concern, we have revised the manuscript to clarify baseline exclusion in greater detail and to interpret findings involving I70.0 as exploratory.

Specifically, participants with any available evidence of aortic disease at or before baseline were excluded from the relevant analyses. In UKB, prevalent aortic disease was identified from linked hospital inpatient diagnosis records (Fields 41270 and 41280), and aortic-related operative procedure records (Fields 41272 and 41282), with primary care records and self-reported history additionally considered where available and if included in the analytic pipeline. In CKB, prevalent aortic disease was identified using baseline survey information and linked electronic health records, including disease registries, and health insurance-based hospital records. Participants with any pre-baseline record of I70.0, I71.0–I71.9, M31.4, or corresponding aortic procedure codes were excluded.

We also performed additional sensitivity analyses using a clinically specific aortic dissection and aneurysm endpoint restricted to ICD-10 codes I71.0–I71.9, excluding both I70.0 and M31.4 (Supplementary Table S13). In UKB, 21 chronic conditions remained associated with this clinically specific endpoint after FDR correction, with the strongest associations observed for coronary heart disease, hypertension, atrial fibrillation, peripheral vascular disease, heart failure, and COPD. In CKB, 10 chronic

conditions remained associated after FDR correction, namely hypertension, coronary heart disease, chronic liver disease, stroke/TIA, chronic kidney disease, cancer, heart failure, RA-OIP-SCTD, COPD, and treated dyspepsia. Thus, although the number of associated conditions was reduced in CKB after excluding I70.0, the retained associations still represented a core set of major cardiovascular and systemic conditions.

To further reduce the possibility that undiagnosed prevalent aortic disease was misclassified as incident disease, we additionally conducted a 2-year lag sensitivity analysis excluding aortic disease events occurring within the first 2 years after baseline (Supplementary Table S14). The overall pattern of association remained similar in both cohorts, supporting the robustness of the findings.

We have revised the Methods, Results, Discussion, and Limitations sections accordingly.

Methods

Page 23, line 529

For each of the 42 chronic conditions, we excluded participants with missing covariate data, those lost to follow-up, those with evidence of prevalent aortic disease at or before baseline, and those whose recorded onset of aortic disease preceded the corresponding chronic condition. To establish the absence of aortic disease at baseline, prevalent aortic disease was defined as any record of aortic disease before or at the baseline assessment in the available cohort-specific data sources. In UKB, these sources included hospital inpatient diagnosis records (Fields 41270 and 41280), aortic-related operative procedure records (Fields 41272 and 41282); primary care records and self-reported medical history were additionally considered where available and if included in the analytic pipeline. In CKB, prevalent aortic disease was identified using baseline survey information and linked electronic health records, including disease registries, and health insurance-based hospital records. Participants with any pre-baseline record of atherosclerosis of aorta (I70.0), aortic dissection and aneurysm (I71.0–I71.9), inflammatory aortic arch syndrome (M31.4), or corresponding aortic procedure codes were excluded from the relevant analyses.

Page 30, line 719

Third, because atherosclerosis of aorta coded as I70.0 may capture asymptomatic, incidental, or previously existing aortic atherosclerosis detected during clinical care, findings involving I70.0 were interpreted cautiously and considered exploratory. We therefore performed an additional sensitivity analysis using a clinically specific aortic dissection and aneurysm endpoint restricted to I71.0–I71.9, excluding atherosclerosis of aorta (I70.0) and inflammatory aortic arch syndrome (M31.4). Fourth, to further minimize the possibility that undiagnosed prevalent aortic disease was misclassified as incident disease, we performed a 2-year lag sensitivity analysis by excluding aortic disease events occurring within the first 2 years after baseline.

Results

Page 14, line 307

To address potential outcome misclassification related to administratively coded aortic atherosclerosis, we repeated the main analyses using a clinically specific aortic aneurysm/dissection endpoint restricted to I71.0–I71.9, excluding atherosclerosis of aorta (I70.0) and inflammatory aortic arch syndrome (M31.4). In UKB, 21 chronic conditions remained associated with the clinically specific endpoint after FDR correction, with the strongest associations observed for coronary heart disease, hypertension, atrial fibrillation, peripheral vascular disease, heart failure, and COPD (**Supplementary Table S13**). In CKB, 10 chronic conditions remained associated after FDR correction, namely hypertension, coronary heart disease, chronic liver disease, stroke/TIA, chronic kidney disease, cancer, heart failure, RA-OIP-SCTD, COPD, and treated dyspepsia. Although the number of associated conditions was reduced compared with the broad composite endpoint, the retained associations represented a core set of major cardiovascular and systemic conditions, supporting the robustness of the main conclusions.

I In a 2-year lag sensitivity analysis excluding aortic disease events occurring within the first 2 years after baseline, the overall pattern of association remained similar (**Supplementary Table S14**). These findings suggest that the observed associations were unlikely to be fully explained by undiagnosed prevalent aortic disease at baseline.

Discussion

Page 17, line 398

Part of this between-cohort heterogeneity may also reflect the outcome definition used for the broad composite endpoint. Because atherosclerosis of aorta coded as

I70.0 may represent asymptomatic plaque, incidental imaging findings, or previously existing disease detected during routine clinical care, the broader administratively coded composite endpoint should be interpreted with caution. Importantly, when we repeated the analyses using a clinically specific endpoint restricted to aortic dissection and aneurysm diagnoses (I71.0–I71.9), the overall pattern of association remained largely unchanged in UKB and persisted for a core set of conditions in CKB, including hypertension, coronary heart disease, stroke/TIA, chronic kidney disease, chronic liver disease, heart failure, RA-OIP-SCTD, COPD, cancer, and treated dyspepsia. The larger attenuation observed in CKB suggests that the broad composite endpoint in that cohort was more strongly influenced by I70.0 coding. Nevertheless, the persistence of associations for major cardiovascular and systemic conditions supports the robustness of the central conclusion that chronic disease burden is associated with clinically defined aortic dissection and aneurysm outcomes.

Limitations

<u>Page 20, line 476</u>

Although participants with any available evidence of prevalent aortic disease at or before baseline were excluded and a 2-year lag sensitivity analysis was conducted, undiagnosed prevalent aortic disease may still have been misclassified as incident disease. This concern is particularly relevant to atherosclerosis of aorta coded as I70.0, which may represent asymptomatic, incidental, or pre-existing aortic disease detected during clinical care. Accordingly, findings involving I70.0 were interpreted as exploratory, and additional analyses using a clinically specific aortic dissection and aneurysm endpoint restricted to I71.0–I71.9 were performed to assess robustness.

Table S13. Sensitivity analysis of the association between chronic conditions and incident aortic dissection and aortic aneurysm in UK Biobank and China Kadoorie Biobank

Condition	UK Biobank		China Kadoorie Biobank	
	HR (95%CI)	FDR *P* value	HR (95%CI)	FDR *P* value
Hypertension	3.27 (3.03, 3.52)	<0.01	4.24 (3.71, 4.85)	<0.01
Painful condition	0.89 (0.52, 1.54)	0.84	0.47 (0.12, 1.90)	0.55
Cancer	1.18 (1.11, 1.26)	<0.01	1.48 (1.31, 1.67)	<0.01
Asthma	1.20 (1.09, 1.32)	<0.01	1.30 (0.97, 1.75)	0.28
Treated dyspepsia	1.29 (1.12, 1.49)	<0.01	2.60 (1.74, 3.87)	<0.01
Coronary heart disease	3.34 (3.12, 3.57)	<0.01	3.19 (2.83, 3.59)	<0.01
Thyroid disorders	1.38 (1.23, 1.54)	<0.01	0.95 (0.74, 1.23)	0.95
Diabetes	1.02 (0.93, 1.12)	0.85	1.12 (0.98, 1.28)	0.30
Depression	1.14 (1.01, 1.28)	0.07	0.94 (0.39, 2.27)	0.98
Psoriasis or eczema	0.98 (0.77, 1.24)	0.95	0.66 (0.16, 2.64)	0.84
Prostate disorders	0.83 (0.67, 1.02)	0.13	0.69 (0.40, 1.19)	0.40
RA-OIP-SCTD	1.15 (1.05, 1.25)	0.01	1.62 (1.38, 1.90)	<0.01
Stroke and TIA	1.51 (1.38, 1.66)	<0.01	2.43 (2.16, 2.73)	<0.01
Osteoporosis	1.21 (1.04, 1.42)	0.03	1.15 (0.89, 1.47)	0.55
COPD	1.78 (1.61, 1.96)	<0.01	1.46 (1.27, 1.67)	<0.01
Migraine	1.55 (1.22, 1.96)	<0.01	2.15 (1.02, 4.53)	0.16
Irritable bowel syndrome	1.35 (1.13, 1.62)	<0.01	0.72 (0.18, 2.87)	0.90
Glaucoma	0.85 (0.72, 0.99)	0.07	0.92 (0.53, 1.59)	0.96
Diverticular disease of intestine	1.10 (1.01, 1.19)	0.05	1.55 (0.83, 2.89)	0.40
ANSS	1.11 (0.97, 1.27)	0.18	1.27 (0.86, 1.86)	0.48

Atrial fibrillation	2.53 (2.35, 2.73)	<0.01	0.96 (0.72, 1.27)	0.96
Inflammatory bowel disease	1.13 (0.91, 1.40)	0.38	0.30 (0.04, 2.15)	0.48
Epilepsy	1.08 (0.86, 1.36)	0.65	0.80 (0.40, 1.59)	0.82
Chronic sinusitis	1.02 (0.75, 1.37)	0.96	0.69 (0.41, 1.14)	0.38
Endometriosis	0.74 (0.39, 1.43)	0.49	0.58 (0.08, 4.14)	0.86
Pernicious anaemia	1.29 (0.88, 1.89)	0.26	2.54 (0.36, 18.05)	0.63
Meniere’s disease	1.03 (0.61, 1.74)	0.96	1.61 (0.86, 3.02)	0.37
Bronchiectasis	1.36 (1.11, 1.67)	0.01	1.02 (0.68, 1.53)	0.98
Chronic fatigue syndrome	1.23 (1.03, 1.47)	0.04	0.96 (0.58, 1.60)	0.98
Peripheral vascular disease	2.47 (2.20, 2.78)	<0.01	0.67 (0.09, 4.75)	0.94
Schizophrenia or BD	0.96 (0.65, 1.41)	0.95	0.53 (0.13, 2.11)	0.63
Parkinson's disease	0.98 (0.75, 1.27)	0.95	0.81 (0.46, 1.44)	0.79
Chronic kidney disease	1.53 (1.38, 1.70)	<0.01	2.63 (2.26, 3.07)	<0.01
Multiple sclerosis	1.55 (0.95, 2.53)	0.13	/	0.98
Viral hepatitis	1.61 (0.93, 2.78)	0.13	0.62 (0.33, 1.15)	0.37
Chronic liver disease	1.21 (1.05, 1.40)	0.02	2.47 (2.19, 2.78)	<0.01
Heart failure	2.38 (2.17, 2.61)	<0.01	1.63 (1.40, 1.90)	<0.01
Alcohol problems	1.35 (1.15, 1.58)	<0.01	/	0.98
Treated constipation	1.21 (1.08, 1.34)	<0.01	0.99 (0.63, 1.57)	0.98
Polycystic ovary	/	0.96	/	/
Anorexia or bulimia	2.81 (0.70, 11.26)	0.21	/	0.98
OPSM	/	0.96	/	0.98

Table S14. Sensitivity analysis of the associations between chronic conditions and incident aortic disease after excluding events occurring within the first 2 years of follow-up in UK Biobank and China Kadoorie Biobank

Condition	UK Biobank		China Kadoorie Biobank	
	HR (95%CI)	FDR *P* value	HR (95%CI)	FDR *P* value
Hypertension	3.28 (3.04, 3.54)	<0.01	3.11 (2.92, 3.32)	<0.01
Painful condition	0.94 (0.55, 1.63)	0.92	0.50 (0.22, 1.12)	0.16
Cancer	1.23 (1.15, 1.32)	<0.01	1.71 (1.61, 1.82)	<0.01
Asthma	1.22 (1.11, 1.35)	<0.01	1.76 (1.54, 2.00)	<0.01
Treated dyspepsia	1.34 (1.16, 1.55)	<0.01	2.17 (1.71, 2.76)	<0.01
Coronary heart disease	3.39 (3.16, 3.63)	<0.01	3.54 (3.34, 3.76)	<0.01
Thyroid disorders	1.40 (1.25, 1.56)	<0.01	1.49 (1.34, 1.65)	<0.01
Diabetes	1.08 (0.99, 1.19)	0.13	1.69 (1.59, 1.80)	<0.01
Depression	1.18 (1.04, 1.33)	0.01	1.54 (1.07, 2.23)	0.04
Psoriasis or eczema	1.07 (0.85, 1.36)	0.65	1.09 (0.62, 1.92)	0.83
Prostate disorders	0.83 (0.66, 1.03)	0.13	0.94 (0.70, 1.26)	0.74
RA-OIP-SCTD	1.19 (1.09, 1.30)	<0.01	1.55 (1.43, 1.68)	<0.01
Stroke and TIA	1.59 (1.45, 1.75)	<0.01	3.36 (3.16, 3.57)	<0.01
Osteoporosis	1.26 (1.07, 1.47)	0.01	1.58 (1.43, 1.74)	<0.01
COPD	1.86 (1.69, 2.05)	<0.01	1.63 (1.52, 1.75)	<0.01
Migraine	1.66 (1.31, 2.09)	<0.01	1.72 (1.17, 2.53)	0.01
Irritable bowel syndrome	1.41 (1.18, 1.69)	<0.01	0.80 (0.42, 1.54)	0.62
Glaucoma	0.88 (0.75, 1.03)	0.18	1.21 (0.96, 1.53)	0.19
Diverticular disease of intestine	1.14 (1.05, 1.23)	<0.01	1.70 (1.29, 2.25)	<0.01
ANSS	1.18 (1.02, 1.33)	0.04	1.41 (1.18, 1.67)	<0.01

Atrial fibrillation	2.61 (2.42, 2.82)	<0.01	1.04 (0.90, 1.20)	0.70
Inflammatory bowel disease	1.18 (0.95, 1.47)	0.18	1.19 (0.76, 1.87)	0.62
Epilepsy	1.16 (0.93, 1.46)	0.25	0.62 (0.41, 0.92)	0.04
Chronic sinusitis	1.08 (0.80, 1.45)	0.73	1.32 (1.11, 1.58)	<0.01
Endometriosis	0.82 (0.44, 1.53)	0.65	0.78 (0.39, 1.56)	0.62
Pernicious anaemia	1.30 (0.88, 1.91)	0.25	3.02 (1.26, 7.26)	0.03
Meniere's disease	0.99 (0.57, 1.70)	0.96	1.11 (0.77, 1.60)	0.68
Bronchiectasis	1.36 (1.11, 1.68)	0.01	1.39 (1.16, 1.65)	<0.01
Chronic fatigue syndrome	1.31 (1.20, 1.57)	<0.01	1.08 (0.84, 1.40)	0.64
Peripheral vascular disease	2.56 (2.27, 2.88)	<0.01	0.42 (0.11, 1.69)	0.34
Schizophrenia or BD	0.96 (0.65, 1.42)	0.92	0.67 (0.37, 1.20)	0.29
Parkinson's disease	1.01 (0.77, 1.32)	0.96	0.97 (0.75, 1.26)	0.87
Chronic kidney disease	1.63 (1.48, 1.81)	<0.01	2.10 (1.93, 2.29)	<0.01
Multiple sclerosis	1.69 (1.05, 2.73)	0.05	2.09 (0.52, 8.37)	0.43
Viral hepatitis	1.55 (0.88, 2.74)	0.18	1.11 (0.87, 1.40)	0.57
Chronic liver disease	1.31 (1.14, 1.51)	<0.01	3.87 (3.66, 4.09)	<0.01
Heart failure	2.54 (2.31, 2.79)	<0.01	1.45 (1.34, 1.56)	<0.01
Alcohol problems	1.46 (1.24, 1.70)	<0.01	1.09 (0.27, 4.38)	0.92
Treated constipation	1.28 (1.15, 1.42)	<0.01	1.16 (0.93, 1.44)	0.31
Polycystic ovary	/	0.96	/	/
Anorexia or bulimia	2.90 (0.72, 11.60)	0.18	0.51 (0.07, 3.61)	0.62
OPSM	/	0.96	/	0.96

Reviewer #3:

Thank you for addressing the previous comments.

Response: We sincerely thank the reviewer for their constructive comments and are pleased that our revisions have addressed the previous concerns.

Response to Reviewers

Manuscript ID: [NCOMMS-25-95985B]

Reviewer #2:

The authors made revisions based on reviewers comments. Thank you.

Response: We sincerely thank the reviewer for the positive comment and for acknowledging our revisions. We appreciate the reviewer's time and constructive feedback throughout the review process.
